# Long-term outcomes of ultrasound-detected hepatic steatosis in primary care: a record linkage study

**DOI:** 10.1136/bmjopen-2026-124118

**Published:** 2026-11-06

**Authors:** Richard J Lilford, Felicity Evison, Katharine Reeves, Magdalena Skrybant, Peter J Chilton, Timothy P Hofer, Samuel I Watson

**Affiliations:** 1 Applied Health Sciences, University of Birmingham, Birmingham, UK; 2 Health Informatics, University Hospitals Birmingham NHS Foundation Trust, Birmingham, UK; 3 Medical School, University of Michigan, Ann Arbor, Michigan, USA

**Keywords:** Hepatology, Primary Care, Hospitalization

## Abstract

**Abstract:**

**Objectives:**

To determine the long-term outcomes of patients with and without ultrasound-detected liver steatosis in primary care to inform guidelines for management of the common finding of steatosis in primary care.

**Design:**

A record-linkage cohort study with 15-year follow-up.

**Setting:**

11 English primary care practices that previously participated in the Birmingham and Lambeth Liver Evaluation Testing Strategies (BALLETS) study.

**Participants:**

A total of 1215 adults with abnormal liver function tests who underwent an intensive set of diagnostic studies, including ultrasound.

**Primary and secondary outcome measures:**

The primary liver outcome was death due to liver disease and/or hospital admission with a primary diagnosis of liver disease. Secondary outcomes were hospital admission where liver disease was recorded as a secondary diagnosis, and outpatient attendance under the care of a liver specialist consultant.

**Results:**

We followed up 760 patients with no steatosis and 455 with steatosis. The unadjusted HR for the primary outcome over 15 years was 1.97 (95% CI 1.23 to 3.01). After making adjustment for age, sex, alcohol intake and Body Mass Index, the probability of the primary liver outcome was 7.7 with no steatosis, vs 14.0 with steatosis—a difference of 6.3% (95% CI 3.2 to 11.2). This relative risk difference was stable over 5, 10 and 15 years. The presence of high Body Mass Index only slightly increased the association between steatosis and liver disease, but the association was more strongly influenced by drinking over the recommended alcohol level.

**Conclusion:**

Over a 15-year follow-up period, the risk of liver disease is approximately doubled (14% vs 7.7%) if steatosis is present.

**STRENGTHS AND LIMITATIONS OF THIS STUDY**

- Successfully linked National Health Service (NHS) numbers to Hospital Episode Statistics and death registries for long-term longitudinal tracking that minimised attrition bias over an extended follow-up period.
- Isolating the non-specific cohort provided direct clinical utility for managing common, incidental findings in primary care.
- The Bayesian survival model was thoroughly verified using posterior predictive checks and Schoenfeld residuals to ensure robust risk estimation.
- Because all participants had baseline abnormal liver function tests, the observed liver outcome rates may be biased upward compared with the general population.
- The reliance on historical ultrasound equipment and multiple sonographers may have introduced inherent inter-observer variability in steatosis diagnosis.

## Introduction

 Accumulation of fat in the liver is caused mainly by metabolic syndrome, alcohol excess or both.^1 2^ It is common and can be detected by ultrasound examination of the upper-right quadrant of the abdomen.^3^ A recent Lancet ‘Seminar’ article recommends the term ‘steatosis of the liver’ to describe this finding, rather than ‘fatty liver’.^4^ Steatosis can progress to more severe diseases; first to steatohepatitis and then to established cirrhosis. There is good evidence that the incidence of cirrhosis has risen over the last few decades and that it is linked to both diet and alcohol.^5 6^ However, as common as severe liver disease is, and notwithstanding its increasing prevalence, it remains rare relative to the prevalence of steatosis which affects around one-third of the adult population, according to recent systematic reviews.^1 2^ It is important therefore to provide scientific evidence regarding the magnitude of the risk of progression since the recommendation has been made that all people with ultrasound-detected steatosis identified in primary care should be investigated by means of serial blood tests and scans of the liver.^7^

Our systematic review (see online supplemental material 1) shows that previous cohort studies are based on populations with steatosis recruited in specialist care (mostly investigated by liver biopsy or liver elastography)^8^ and followed up for a median of 4 years. There are no studies with long-term follow-up of patients with steatosis recruited in primary care, resulting in opinions that range from ‘NAFLD [non-alcoholic fatty liver disease] – sounding the alarm on a silent epidemic,’^9^ to ‘too much medicine: over-diagnosis and overtreatment of NAFLD.’^10^

To shed some light on this issue, we followed up patients recruited to a previous study 15–19 years ago. This previous study was commissioned by the National Institute for Health and Care Research (NIHR) to investigate the causes of abnormal liver function tests (LFTs) in primary care—the Birmingham and Lambeth Liver Evaluation Testing Strategies (BALLETS) study.^11^ In that study, patients were recruited on the basis that they had one or more abnormal analytes on a liver test panel. All consenting patients underwent extensive tests, including liver ultrasound, to determine the cause of the abnormal test. We found that few of these patients with abnormal liver enzymes had a well-characterised specific disease like viral hepatitis, haemochromatosis, primary biliary cholangitis or alcoholic liver disease.^12^ However, a large proportion of those that did not have one of these diseases had steatosis on ultrasound examination.^11^

Here, we report a long-term follow-up of the whole BALLETS cohort to observe outcomes for patients in primary care with no specific liver disease, but at least one abnormal analyte on their LFT, according to whether they did or did not have steatosis on ultrasound scan. We excluded patients where the original study identified a specific, well-characterised liver disease, such as chronic viral hepatitis and primary biliary cholangitis, because the prevalence of these conditions varies from one population to another and because the primary care practitioner will often have, or can acquire, this information (see Discussion). We examined risk factors that may be associated with rapid progress of steatosis cases, including the degree of fatty infiltration, Body Mass Index (BMI) and alcohol intake that were measured at baseline.

To our knowledge, this is the largest cohort study of patients with ultrasound-detected steatosis recruited in primary care and followed up for liver-related outcomes for over 15 years. The combination of one or more abnormal analytes and steatosis on ultrasound in a patient who is not a heavy drinker is a common scenario in primary care. Our results will be most directly referable to such a patient but also provide:

1. An upper limit for the probability of serious liver disease for people with an ultrasound diagnosis of steatosis, but no abnormal liver test result (on the plausible assumption that people with steatosis and abnormal liver enzymes have a higher risk of progression than those with steatosis but normal enzymes).
2. Evidence to inform the need for ultrasound in people with (mildly) abnormal LFTs.

## Methods

The protocol for this follow-on study was published online in February 2022: https://osf.io/2tybj (doi: 10.17605/OSF.IO/2TYBJ), and we have reported according to Strengthening the Reporting of Observational Studies in Epidemiology (STROBE) criteria (online supplemental material 2).

### The BALLETS study

A detailed account of the original BALLETs study can be found elsewhere.^11^ Briefly, the BALLETS study was a prospective study of people in primary care who had abnormal LFTs. These tests consist of a panel of six to eight analytes (depending on the laboratory). 11 primary care practices in England (eight in Birmingham, three in the Lambeth area in London) contributed to the study. To be eligible, patients had to have no known liver disease at the time of entry into the study, and at least one abnormal analyte on their most recent LFT panel. Recruitment took place between 2005 and 2008. Eligible patients (n=1290) attended for a visit to primary care within 6 weeks of their LFT where clinical details were recorded: age, sex, ethnic group, country of birth, reason for blood test, alcohol and medication history, and BMI. A blood sample was taken to repeat the LFT and to test for the specific diseases listed in online supplemental material 3. An ultrasound scan of the liver was performed by one of five trained sonographers (50 scans were reviewed by a liver radiologist for quality control purposes). The study was designed to examine the relationship between different liver test results and one of the diseases in online supplemental material 3 or a space-occupying lesion.

### Generation of the cohort

Based on all data available to the team, patients were grouped into four primary categories:

1. Hepatocellular disease.
2. Hepatobiliary disease.
3. ‘Tumour’, meaning any space-occupying lesion on liver ultrasound.
4. Non-specific:
  - With steatosis as discerned on ultrasound.
  - Without steatosis.

If a patient with one of the first three category diseases also had steatosis, they were placed in one of these categories and not in the steatosis category (ie, not in 4a). The original study findings have been reported elsewhere.^11 12^

Here, we are concerned with the fourth group above, which is made up of the majority of patients who did not have serious, specific disease of, or in, their liver. Only 53 of the original BALLETS cohort of 1290 patients (4.1%) were assigned to specific categories (1–3 above). It is the remaining non-specific category (1237 patients) that is the focus of this study.

### Data linkage

Participants from the original BALLETS study were matched using their National Health Service (NHS) number to two databases: the Hospital Episode Statistics (HES) and the Civil Registrations of Death (Deaths). We extracted from the HES database the reason for any hospital attendances, and from the Deaths database we obtained first, deaths of any cause and second, the subgroup of deaths where liver disease was the main or a contributory cause. Further details and data-flow diagrams are provided in the protocol (https://osf.io/2tybj).

### Outcomes

Our primary outcome is the binary composite of: (1) hospital admission with a primary diagnosis of liver disease or the patient was in the care of a liver consultant (henceforth ‘primary diagnosis of liver disease’); and/or (2) death where liver disease is mentioned as a cause (ie, the main or a contributing cause). Our purpose in combining the above variables was to identify cases where it was likely that a disease of the liver was present while not narrowing selection down to the degree that we would miss cases where ultrasound was prognostic.

Although our primary diagnosis concerned admissions where liver disease was primary, we also conducted a ‘secondary’ analysis where we examined associations between steatosis and: (1) admissions where liver disease was a secondary cause; and (2) outpatient department attendance under the care of a liver consultant. The purpose of this secondary analysis was to examine for consistency between our selected outcome (admissions with a primary diagnosis) and alternative outcomes that might have been chosen to examine the same underlying causal pathway (admission including secondary diagnoses and cases seen only in the outpatient department). Statistical analyses were done to compare the magnitude of effect sizes with the primary analysis.

### Coding liver disease (HES)

We recorded all admissions where liver disease was the main or contributing cause. The full list of diseases of the liver is given in online supplemental material 4.

### Statistical methods

We used a Bayesian survival analysis approach to analysing the data. The exposure time was months from the ultrasound in the original BALLETS study ultrasound where it was determined whether the patient had steatosis or not. Patients who died from non-liver causes were right censored at death. We examined whether a proportional hazard assumption was reasonable using graphical comparisons of survival and log cumulative hazard functions.^13^ We also calculated and plotted the Schoenfeld residuals.^14^ These analyses are reported in online supplemental material 5. All analyses supported the use of proportional hazards.

For our model, there were several plausible alternatives for the baseline hazard function specification (exponential, Weibull and semiparametric—with basis functions 3 and 5). We compared between these specifications using graphical posterior predictive model checks and the leave-one-out cross-validation (LOO-CV) criterion.^15^ Covariates used in adjustment included standardised age and its square, sex, BMI standardised and its square, and alcohol usage above the contemporary recommended units. We fit unadjusted models, models adjusted for age and sex alone, and then including BMI and alcohol (to examine the association between steatosis and alcohol, net of these factors).

We used very weakly informative prior distributions provided a degree of regularisation but little information on parameter values beyond this. Specifically, we used an N(0, 5^2^) prior for log-hazard ratio intervals. From the estimated survival models, we obtained samples from the posterior predictive distribution of time to event among the whole cohort. We also report predicted probabilities among the subgroup with BMI >30. We estimated the probability of liver outcome by liver status or degree and number of years post-diagnosis; we also reported HRs comparing steatosis to no-steatosis. The small number with missing BMI or alcohol information (recorded in data-summary tables) were excluded from the fully adjusted model.

## Patient and public involvement

We sought feedback from six public contributors about potential ethical implications of longer-term follow-up of patients from the original BALLETS study. From this, we concluded there were no preclusive ethical issues.

## Results

### Analysis

#### Summary statistics

The initial BALLETS cohort comprised 1290 patients who had at least one abnormal LFT. We then excluded those with serious liver disease (classified as 1a (n=30), 1b (n=13) or 2 (n=10)); patients whose data we were unable to link due to errors or missing data in the items used to link to HES data (n=11); and patients who had not had a liver scan or whose liver could not be fully visualised (n=11). This left a final cohort of 1215 patients. Of these, 455 (37.4%) were identified as having steatosis at baseline, of which 240 (52.7%) were categorised by the sonographer on the basis of liver texture as having mild disease, 171 (37.6%) as moderate and 44 (9.7%) as marked. The median follow-up time was 15.3 years (95% CI 15.2 to 15.4) for patients without steatosis at baseline; 15.6 years (95% CI 15.4 to 15.7) for mild; 15.3 (95% CI 15.2 to 15.4) years for moderate; and 15.4 years (95% CI 15.2 to 15.6) for marked steatosis.

### Baseline characteristics of the steatosis and no steatosis groups

A summary of cohort demographic characteristics is shown in table 1. Patients with steatosis were slightly younger at baseline than those without (mean (SD): 56.3 (12.7) vs 58.6 (16.7) years), they were more likely to be male (62.9% vs 52.5%), and less likely to be from a Black ethnic background (2.2% vs 7.1%). Most patients with steatosis were classified as overweight (25–29.99 kg/m^2^) or obese (>30 kg/m^2^) based on their BMI at the first follow-up visit (90.6%), compared with 65.9% of patients without steatosis. A higher proportion of people with steatosis drank above the recommended alcohol units per week (14 units/week) at the time of follow-up (35.8% vs 26.4%). Patients with steatosis were less likely to have high co-morbidity scores. However, breakdown of co-morbidities (online supplemental material 6) shows that they were more likely to be diagnosed with diabetes, hypertension and depression. Breakdown by individual liver function analytes and reasons for testing (online supplemental material 7) shows that the cohort with steatosis was more likely to have abnormal aspartate aminotransferase (AST) or alanine aminotransferase (ALT) results (among the panel of LFTs) than the cohort without steatosis. The steatosis cohort was also more likely to have had the initial tests done as part of routine testing, for example, annual diabetes checks.

**Table 1:** Patient demographics by liver steatosis status

	Steatosis		
Demographics	Not present, n=760	Present, n=455	Total n=1215
Age at baseline, mean (SD)	58.6 (16.7)	56.3 (12.7)	57.7 (15.3)
Age (years)			
≤34	75 (9.9%)	24 (5.3%)	99 (8.1%)
35–44	98 (12.9%)	58 (12.7%)	156 (12.8%)
45–54	108 (14.2%)	114 (25.1%)	222 (18.3%)
55–64	171 (22.5%)	132 (29.0%)	303 (24.9%)
65–74	155 (20.4%)	97 (21.3%)	252 (20.7%)
75+	153 (20.1%)	30 (6.6%)	183 (15.1%)
Sex recorded			
Female	361 (47.5%)	169 (37.1%)	530 (43.6%)
Male	399 (52.5%)	286 (62.9%)	685 (56.4%)
Ethnic group			
White	605 (79.6%)	389 (85.5%)	994 (81.8%)
South Asian	52 (6.8%)	31 (6.8%)	83 (6.8%)
Black	54 (7.1%)	10 (2.2%)	64 (5.3%)
Other	24 (3.2%)	14 (3.1%)	38 (3.1%)
Not known	25 (3.3%)	11 (2.4%)	36 (3.0%)
BMI*			
<20	34 (4.5%)	3 (0.7%)	37 (3.0%)
20–24.99	206 (27.1%)	33 (7.3%)	239 (19.7%)
25–29.99	286 (37.6%)	151 (33.2%)	437 (36.0%)
30+	215 (28.3%)	261 (57.4%)	476 (39.2%)
Not known	19 (2.5%)	7 (1.5%)	26 (2.1%)
Alcohol units consumed*			
0	341 (44.9%)	184 (40.4%)	525 (43.2%)
1–14	213 (28.0%)	107 (23.5%)	320 (26.3%)
15–29	92 (12.1%)	53 (11.6%)	145 (11.9%)
30–49	63 (8.3%)	55 (12.1%)	118 (9.7%)
50–99	37 (4.9%)	42 (9.2%)	79 (6.5%)
100+	9 (1.2%)	13 (2.9%)	22 (1.8%)
Not known	5 (0.7%)	1 (0.2%)	6 (0.5%)
Alcohol risk†			
High	170 (22.5%)	139 (30.6%)	309 (25.6%)
Low	585 (77.5%)	315 (69.4%)	900 (74.4%)
Unknown	5	1	6
Elixhauser score			
<0	79 (10.4%)	68 (14.9%)	147 (12.1%)
0	392 (51.6%)	245 (53.8%)	637 (52.4%)
1–4	104 (13.7%)	63 (13.8%)	167 (13.7%)
5+	185 (24.3%)	79 (17.4%)	264 (21.7%)
Charlson score			
0	402 (52.9%)	203 (44.6%)	605 (49.8%)
1–2	279 (36.7%)	209 (45.9%)	488 (40.2%)
3+	79 (10.4%)	43 (9.5%)	122 (10.0%)

* BMI and alcohol units consumed over past 6 months are taken from the first follow-up visit.

† Patients were classified as high risk if they drank more than the recommended number of units at the time of the study, and patients classed as low risk are people who drank fewer than the recommended number of units at the time of the original BALLETS study.

BMI, Body Mass Index.

### Primary liver outcome

Table 2 shows patient outcomes by steatosis diagnosis, overall and by degree (grades 1–3). 90 patients had at least one admission for liver disease (defined as admission with a liver diagnosis code in the primary position or ‘under a liver consultant’). An additional seven patients died with liver disease as a cause. Thus, 97 patients experienced the primary outcome, of whom 53 (11.6%) were patients with steatosis and 44 (5.7%) were those without steatosis—a difference of 5.9 percentage points. A breakdown of the International Classification of Diseases, 10th Revision (ICD-10) disease codes across the patients with the primary liver outcome is given in online supplemental material 8. The full disbursement of the 1215 patients across ‘exposures’ (steatosis/no steatosis), admissions (yes/no), dead or alive, and liver disease (yes/no) is shown in online supplemental material 9.

**Table 2:** Outcomes by steatosis (overall and by degree (1–3))

			Alive at census (n=814)		Died (n=401)				Primary liver outcome
					Died with liver disease (n=20)		Died not with liver disease (n=381)		
			Never in-patient with diagnosis of liver diseases	≥1 in-patient admission for liver disease	Admitted for liver disease	Not admitted for liver disease	Admitted for liver disease	Not admitted for liver disease	
Sub-objective A	No steatosis (n=760)		468	23	≤5	≤5	15	250	44 (5.7%)
Sub-objective B	Grade of steatosis	1-3 (n=455)	294	29	9	≤5	12	110	53 (11.6%)
		1 (n=240)	152	14	≤5	≤5	5	60	25 (10.4%)
		2 (n=171)	114	15	≤5	≤5	≤5	30	25 (14.6%)
		3 (n=44)	28	0	≤5	0	0	15	≤5

NB. Small numbers (≤5) have been suppressed; where a small number could be reverse-calculated other cells have been rounded to the nearest 5. Note that these changes affect the presentation of quantities in table 2, not the analyses carried out. Admission for liver disease is any admission with a liver diagnosis in the primary position or where the consultant was contracted under a liver specialty.

### Other liver outcomes

Findings for patients who were admitted with a secondary diagnosis of liver disease or those who attended the outpatient department for possible liver disease are presented in online supplemental material 10. Here, it can be seen that admissions in the secondary diagnosis category, outpatient attendances overall and hospital visits of any type with a liver condition are all higher in the steatosis than not steatosis categories. Only liver outpatient visits in people specifically with no admission were higher in the no steatosis group and here the difference is small and uncertain. No patient was recorded as having a liver transplant during the follow-up period.

### Survival analyses for the primary liver outcome

Panel A of figure 1 shows the unadjusted Kaplan-Meier survival curves for the primary outcome and Panels B and C report predicted survival curves adjusted for age, sex, BMI and alcohol risks. The LOO-CV and Widely Applicable Information Criterion (WAIC) suggested that the semi-parametric models had a slightly better fit (online supplemental material 11); however, the graphical posterior predictive checks indicated that the Weibull models were superior (online supplemental material 12). The results presented here used the Weibull models.

**Figure 1:**
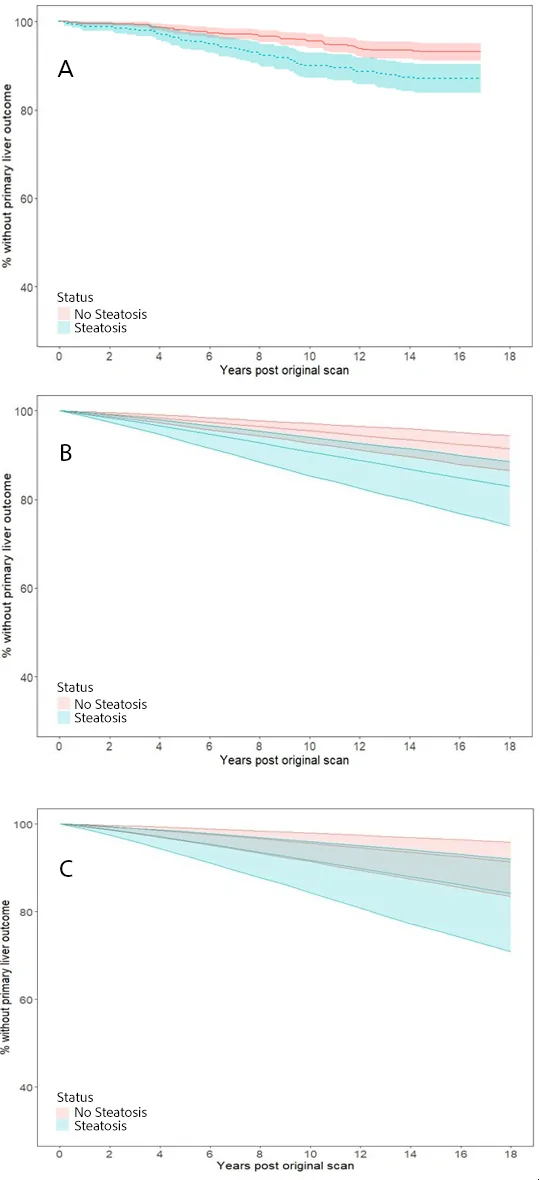
Survival curves for the primary outcome of admission for liver disease or death with liver disease. (A) The unadjusted Kaplan-Meier survival curves. (B) The age, sex-adjusted model. (C) The fully adjusted model.

HRs are presented in table 3. After adjustment for age and sex, there remains an increased risk of experiencing a liver-related endpoint in the steatosis group versus not steatosis group (HR 2.16; 95% CrI 1.38, 3.17). There was little evidence of a relationship between BMI and the probability of the liver outcome, which is also illustrated in table 4. Alcohol intake was relatively more predictive of the liver outcome (HR 1.77; 1.12–2.67).

**Table 3:** HRs and credible limits for primary outcome unadjusted, adjusted for age and sex, and fully adjusted after 15.5 years

	HR (interval)	HR (interval)	HR (interval)
Primary outcome	2.00 (1.33, 2.94)	2.16 (1.38, 3.21)	1.97 (1.23, 3.01)
Standardised age		1.14 (0.89, 1.43)	1.20 (0.92, 1.51)
Standardised age squared		1.05 (0.84, 1.27)	1.10 (0.88, 1.34)
Sex (male)		0.65 (0.43, 0.95)	0.60 (0.38, 0.90)
High alcohol risk			1.77 (1.12, 2.67)
Standardised BMI			1.18 (0.89, 1.55)
Standardised BMI squared			0.93 (0.79, 1.04)

Note that it is not expected that the adjusted HRs will correspond exactly to the differences in absolute rates in table 4.

BMI, Body Mass Index.

**Table 4:** Posterior predicted probability of having a liver outcome (95% CrI) and the difference in probability

Model	5 years			10 years			15 years		
	Net risk of primary outcome (%)		Difference (percentage point)	Net risk of primary outcome (%)		Difference (percentage point)	Net risk of primary outcome (%)		Difference (percentage point)
	No steatosis	Steatosis		No steatosis	Steatosis		No steatosis	Steatosis	
Unadjusted	1.9 (1.1, 2.8)	3.9 (2.8, 5.2)	2.0 (0.6, 3.6)	4.1 (2.8, 5.2)	8.2 (6.7, 10.4)	4.1 (2.4, 6.6)	6.4 (5.0, 8.1)	12.8 (11.0, 15.3)	6.4 (4.0, 9.2)
Adjusted age and sex	2.1 (1.2, 3.5)	4.3 (2.7, 6.9)	2.3 (1.3, 3.6)	4.5 (2.9, 7.3)	9.2 (6.0, 14.7)	4.7 (2.9, 7.5)	7.1 (4.5, 11.2)	14.1 (9.3, 21.7)	7.1 (4.3, 10.9)
Fully adjusted	2.0 (0.9, 4.0)	3.8 (1.8, 7.3)	1.8 (0.7, 3.5)	4.4 (2.1, 8.6)	8.3 (4.1, 15.7)	3.9 (1.8, 7.2)	7.0 (3.3, 13.5)	13.0 (6.5, 24.2)	5.9 (2.9, 10.7)
Fully adjusted, BMI >30*	2.2 (1.1, 4.1)	4.1 (2.1, 7.6)	1.9 (0.8, 3.6)	4.8 (2.5, 9.3)	9.0 (4.8, 16.6)	4.1 (1.9, 7.4)	7.7 (4.1, 14.3)	14.0 (7.7, 25.2)	6.3 (3.2, 11.2)

* Posterior predictive probability among only those with a BMI >30 using the fully adjusted model estimated on the whole dataset.

BMI, Body Mass Index.

Regarding cumulative incidence (table 4), 7.1% of patients with no steatosis had a primary liver outcome, vs 14.1% of patients with steatosis (after adjusting for age and sex). Thus, patients with steatosis had a posterior predicted probability of experiencing a liver related outcome 7.1 (95% CrI 4.3, 10.9) percentage points higher than those without steatosis after 15 years. The difference in predicted probability rises progressively from 2.3 percentage points at 5 years to 7.1 at 15 years, with an approximate twofold difference across the three epochs, as seen in table 4. Reassuringly, the HRs were similar regardless of choice of baseline hazard function (online supplemental material 13).

## Discussion

### Main findings

It is clear that the presence of steatosis on a scan is informative—notwithstanding measurement classification error, the risk of subsequent liver disease is increased about twofold following observation of steatosis. The probability of a liver end-point over ~15 years of follow-up is increased by about seven percentage points, adjusted for age and sex, decreasing to about six percentage points when also adjusted for obesity and alcohol intake. This is likely to reflect the effect of baseline obesity and alcohol on the explanatory variable (steatosis) and the subsequent effects of ongoing excess calorie and alcohol intake. The steatosis cohort was more likely to have had their initial blood test as part of routine checks for conditions like diabetes, rather than presenting with symptoms. This is consistent with the theory that steatosis is a feature of an underlying metabolic syndrome.

### Comparison with other literature

As stated, this is the longest and largest cohort study of steatosis in a primary care population. We find clear evidence of progression of liver disease, but at a slower rate than in a hospital-based population. There were several studies of outcome for steatosis in hospital/specialist populations, which are influenced by spectrum bias,^16^ and which include liver biopsy or, more recently, various tests for liver fibrosis.^8 17 18^ Our results are more consistent with population-based studies comparing mortality rates in patients (diagnosed with what was then called non-alcoholic fatty liver disease) with overall mortality rates. For example, a study of 420 such patients using the Rochester Epidemiology Project records system found a standardised mortality ratio of 1.34 compared with the general population over a mean follow-up of 7.6 years.^19^ Further examples are given in the systematic review cited above.

### Methodological issues, strengths and limitations

Our design is based on our purpose—we imagine a consultation between patient and primary care practitioner, and we try to inform the decision regarding what action, if any, should be taken, contingent on findings from liver ultrasound. A number of factors should be considered in the interpretation of our findings.

1. We considered including cardiovascular disease as an outcome, but decided, given the many other risk factors that clinicians use to assess cardiovascular risk, that the marginal utility of steatosis (or not) is likely low. We did not have Confidentiality Advisory Group (CAG) permission for examination of cardiovascular outcomes.
2. We exclude people with specific diseases at the time of the original diagnosis because the risk of these conditions varies around the world; these conditions can be detected by specific tests; and we wish to inform the decision where the doctor is not concerned about the existence of such a disease, but rather with the risk inherent in steatosis itself. We include a variety of outcome liver codes to inform an overall risk assessment for primary care practitioners, given that aetiologies overlap and classifications are unreliable.
3. The results of our study apply most directly to a population attending primary care, having an LFT test and then receiving at least one abnormal result on the LFT panel. The outcomes, especially liver outcomes, are likely to occur at a much lower rate than among secondary care or biopsy cohorts. However, they will likely be biased upward for people not having an abnormal LFT, not having an LFT test or not ever attending primary care.
4. The ultrasound diagnosis of steatosis is influenced by operator, equipment type and body morphology. However, all of these factors were broadly representative of a primary care context and the prevalence of steatosis is in line with other similar studies.
5. We have considered combining outpatient attendance, inpatient attendance and death into a compound outcome, but decided that this would not be useful given the large difference in utility between death and a single outpatient appointment. At the other extreme, we could have pre-determined an analysis based solely on the presence of steatosis. We did not do so because we thought there might be few cases and because liver diagnoses are not always watertight when more than one factor contributes.
6. We do not believe that referral was prompted by the finding of steatosis during the scan performed as part of this study because the difference in primary outcome is small at 5 years, rising progressively thereafter (table 4), and there is almost no difference in the first 2 years (figure 1, Panel A). Also, we used admission rather than attendance at outpatient’s department to construct our primary outcome in part to avoid thus problem. In the event, similar event rates were recorded across both of these outcome types.
7. Liver diseases may progress slowly, such that many cases may not have become manifest over the range of follow-up now possible for the BALLETS cohort. The database at University Hospitals Birmingham NHS Foundation Trust (UHBFT) will be destroyed (to meet conditions to obtain access to linked data) but we will maintain the original BALLETS data archive at the University of Birmingham so that ethical approval can be sought for re-analysis after a further decade has elapsed.
8. We do not report an overall mortality because the findings are imprecise, but we include these data in online supplemental material 14 so that it may be combined with other data that may be published in the future.
9. Our HR estimand (table 3) is an associative relative effect (the cause-specific HR is defined as the rate conditional on being alive and disease-free). The net risk (table 4) gives a clinically useful measure of the rate of progression (admission or death for liver causes) but by estimating the net risk, meaning in the absence of competing risks, it overstates the absolute probability for people with substantial competing mortality, some of whom would die from a competing cause before a liver outcome occurs. In this cohort, non-liver deaths outnumbered liver outcomes by roughly four to one (381 vs 90–97) so the amount by which the probability is smaller is not trivial when taking competing risks into account. Published comparisons of a similar magnitude of competing events find over-estimates on the order of 15%–20% (relative increase).^20^ For younger and healthier cohorts with lower non-liver mortality, the difference from our net risk estimates would be smaller.

### Implications

The clinical implications must take into account the finding that risk of liver disease increases when steatosis is found on ultrasound, but to a modest degree in absolute (percentage point) terms. That said, steatosis is associated with a doubling in risk at 15 years, and the absolute risk difference is likely to increase over periods longer than 15 years or in patients with higher baseline risk of significant liver disease outcomes. Our findings thus support behavioural interventions and are consistent with a recent Lancet consensus guideline that abnormal analytes on the LFT panel should prompt an upper-right quadrant scan, and that if this is positive for steatosis, should in turn provoke a fibrosis score^4^ (although the guidelines suggest that abnormal liver function tests alone are an indication for fibrosis testing if no other cause can be identified).

## Conclusion

In conclusion, steatosis is not a meaningless observation and approximately doubles the risk (from ~7% to ~14%) of a liver outcome of sufficient severity to be the primary cause of admission (or death) in patients with abnormal liver enzymes. Unlike most other studies, we provide evidence from a primary care population that does not already have classifiable liver disease. We think our findings will be useful for those who produce or update guidelines/algorithms for management of people in primary care with ultrasound evidence of steatosis.

### Transparency declaration

The lead author (the manuscript’s guarantor) affirms that the manuscript is an honest, accurate and transparent account of the study being reported; that no important aspects of the study have been omitted; and that any discrepancies from the study as planned have been explained.

## Supplementary material

10.1136/bmjopen-2026-124118 online supplemental file 1

## Data Availability

Data may be obtained from a third party and are not publicly available. No participant data are available.
