# Supplementary material for: Long-term outcomes of ultrasound-detected hepatic steatosis in primary care: a record linkage study
Source: BMJ Open. 2026 Nov 6;16(11):e124118. doi: 10.1136/bmjopen-2026-124118 (PMC13756320; doi:10.1136/bmjopen-2026-124118)
Supplement: Supplementary file 1 [file bmjopen-16-11-s001.pdf]

Supplementary Materials

Supplementary Material 1: Previous evidence on progression of Non-Alcoholic Fatty Liver Disease (NAFLD)	2
Supplementary Material 2: STROBE Statement—checklist of items that should be included in reports of observational studies	4
Supplementary Material 3: Table: Liver disease (viral, genetic and autoimmune) for which all BALLETS patients were tested	7
Supplementary Material 4: Table: List of Diseases of the Liver and their HES code	8
Supplementary Material 5: Figure: Analyses conducted to assess the proportional hazards assumption	11
Supplementary Material 6: Table: Individual comorbidity results	13
Supplementary Material 7: Table: Results of the analyte performed at baseline by steatosis status, and reason original testing was done	14
Supplementary Material 8: Figure: Frequency ICD10 Coding Among Patients with Primary Liver Outcome	15
Supplementary Material 9: Figure: Description of the status of patients in the cohort.	16
Supplementary Material 10: Secondary liver outcomes	17
Supplementary Material 11: Figure: Graphical posterior predictive model checks for the primary outcome	18
Supplementary Material 12: Table: Goodness of fit results for the different models looking at the primary outcome of admission for liver disease or death due to liver disease.	19
Supplementary Material 13: Table: Hazard Ratios for all models	20
Supplementary Material 14: Overall Survival Analysis (all-cause mortality)	21

Supplementary Material 1. Previous evidence on progression of Non-Alcoholic Fatty Liver Disease (NAFLD)

We carried out a systematic search of the SCOPUS database using the following search strings:

No.	Type	String	Results
#1	Title-Abs-Key	“cohort study” OR “prospective study” OR “longitudinal study” OR "Prognostic study" OR "Follow-up study" OR “Follow up study”	1,721,624
#2	Title-Abs-Key	"Nonalcoholic fatty acid liver disease" OR “Nonalcoholic fatty liver disease”	12,844
#3	-	#1 AND #2	1,495
#4	Key	death OR mortality OR (hospital W/2 admission) OR prognosis	2,249,051
#5	-	#3 AND #4	241
#6	-	Limit #5 to Human	240
#7	-	Limit #6 to English	240
#8	-	Limit #7 to 2005 onwards	238
#9	-	Limit #8 to Reviews	24

Conducted in SCOPUS, 22 April 2021.

We then examined reference lists of retrieved studies and references retrieved from a recent topic-wide reviews in JAMA (1) and BMJ (2) for further studies we may have missed.

Two reviews since 2012 were identified, based on cases of NAFLD diagnosed by means of liver biopsies.(3, 4) The latter review in 2020 included 13 studies (4,428 patients) with a mean follow-up time of six years; ten included clinical outcomes. For example, Singh, et al. found that patients progressed at a mean of one point on a five point scale over 7·7 years.(5) The more severe the pathology, the greater the risk of progression. However, these studies involve highly selected populations warranting an invasive liver biopsy in secondary care, and the results are therefore likely to greatly overestimate outcomes among a clinical population in primary care. More recently, a massive multi-centre study followed-up 16,603 patients who underwent elastography screening of the liver, finding that this test, like liver biopsy, is sensitive and specific in predicting serious liver disease.(6) However, again this study was based on specialist centres.

We also identified two recent studies on the prevalence of NAFLD in general populations – Adams, et al.,(7) and Allen, et al.(8) – along with an older, but famous Dionysus study in Italy.(9) The study by Adams, et al. looked at the natural history of 420 NAFLD patients diagnosed over a 20 year span, with a mean follow-up of 7·6 years, and found higher mortality compared to the general population, with liver-related death being leading cause of mortality. Allen, et al. identified 3,859 NAFLD patients and 15,209 cohorts with a median seven years follow-up as part of the Rochester Epidemiology Project, which used a multistate model. They observed a reduced life expectancy of four years in NAFLD patients, but the impact of NAFLD on cardiovascular events and death was significant only in people without metabolic abnormalities such as dyslipidaemia, hypertension or diabetes.

The BALLETS study was different to the above studies of general population in that the cases were identified in primary care. Two systematic reviews since 2012 included information on prognosis in NAFLD patients who did not have a biopsy diagnosis (populations more similar to the BALLETS cohort). One ranged back to 1965 and included cross-sectional studies. The remaining review (10) reported on six cohort studies. One of these provided prognostic information on only 273 patients,(11) another was restricted to patients who *already* had cardiovascular disease at entry,(12) another included only three years follow-up,(13) while the fourth paper evaluated only cardiovascular outcomes.(14) This left two separate papers, both of which (surprisingly) analysed the *same* data from the US National Health and Nutrition Survey,(15, 16) and these were based on healthy volunteers and examined only mortality outcomes, finding no differences in liver mortality, but with wide confidence limits.

Despite limited information, a number of recommendations for primary care have been made, one suggesting that the term NAFLD should be replaced with MAFLD (Metabolic Associated Fatty Liver Disease).(17) An

important prospective evaluation of primary care referral pathways for patients with NAFLD included 3,012 participants from primary care followed up according to whether or not they were allocated to undergo a scan of the liver to detect fibrosis.(18) This study did not include clinical outcomes.

We have also found papers describing guidelines for clinical practice, one published in the Lancet in 2024.(19)

In conclusion, there are a number of papers based on histological diagnosis of NAFLD and some general population-based studies. There is little solid information on liver-related outcomes in these populations, and still less on outcome for ‘fatty liver’ that might be found in primary care.

References:

1. Rinella ME. Nonalcoholic fatty liver disease: a systematic review. Jama. 2015;313(22):2263-73.
2. Petroni ML, Brodosi L, Bugianesi E, Marchesini G. Management of non-alcoholic fatty liver disease. BMJ. 2021;372:m4747.
3. Pais R, Charlotte F, Fedchuk L, Bedossa P, Lebray P, Poynard T, et al. A systematic review of follow-up biopsies reveals disease progression in patients with non-alcoholic fatty liver. J Hepatol. 2013;59(3):550-6.
4. Taylor RS, Taylor RJ, Bayliss S, Hagström H, Nasr P, Schattenberg JM, et al. Association Between Fibrosis Stage and Outcomes of Patients With Nonalcoholic Fatty Liver Disease: A Systematic Review and Meta-Analysis. Gastroenterology. 2020;158(6):1611-25.e12.
5. Singh S, Allen AM, Wang Z, Prokop LJ, Murad MH, Loomba R. Fibrosis progression in nonalcoholic fatty liver vs nonalcoholic steatohepatitis: a systematic review and meta-analysis of paired-biopsy studies. Clin Gastroenterol Hepatol. 2015;13(4):643-54.e1-9; quiz e39-40.
6. Lin H, Lee HW, Yip TC, Tsochatzis E, Petta S, Bugianesi E, et al. Vibration-Controlled Transient Elastography Scores to Predict Liver-Related Events in Steatotic Liver Disease. Jama. 2024;331(15):1287-97.
7. Adams LA, Lymp JF, St Sauver J, Sanderson SO, Lindor KD, Feldstein A, et al. The natural history of nonalcoholic fatty liver disease: a population-based cohort study. Gastroenterology. 2005;129(1):113-21.
8. Allen AM, Therneau TM, Larson JJ, Coward A, Somers VK, Kamath PS. Nonalcoholic fatty liver disease incidence and impact on metabolic burden and death: A 20 year-community study. Hepatology. 2018;67(5):1726-36.
9. Bellentani S, Tiribelli C, Saccoccio G, Sodde M, Fratti N, De Martin C, et al. Prevalence of chronic liver disease in the general population of northern Italy: the Dionysos Study. Hepatology. 1994;20(6):1442-9.
10. Mahfood Haddad T, Hamdeh S, Kanmanthareddy A, Alla VM. Nonalcoholic fatty liver disease and the risk of clinical cardiovascular events: A systematic review and meta-analysis. Diabetes Metab Syndr. 2017;11 Suppl 1:S209-s16.
11. Fracanzani AL, Tiraboschi S, Pisano G, Consonni D, Baragetti A, Bertelli C, et al. Progression of carotid vascular damage and cardiovascular events in non-alcoholic fatty liver disease patients compared to the general population during 10 years of follow-up. Atherosclerosis. 2016;246:208-13.
12. Wong VW, Wong GL, Yip GW, Lo AO, Limquiaco J, Chu WC, et al. Coronary artery disease and cardiovascular outcomes in patients with non-alcoholic fatty liver disease. Gut. 2011;60(12):1721-7.
13. El Azeem HA, Khalek el SA, El-Akabawy H, Naeim H, Khalik HA, Alfifi AA. Association between nonalcoholic fatty liver disease and the incidence of cardiovascular and renal events. J Saudi Heart Assoc. 2013;25(4):239-46.
14. Hamaguchi M, Kojima T, Takeda N, Nagata C, Takeda J, Sarui H, et al. Nonalcoholic fatty liver disease is a novel predictor of cardiovascular disease. World J Gastroenterol. 2007;13(10):1579-84.
15. Lazo M, Hernaez R, Bonekamp S, Kamel IR, Brancati FL, Guallar E, et al. Non-alcoholic fatty liver disease and mortality among US adults: prospective cohort study. Bmj. 2011;343:d6891.
16. Stepanova M, Younossi ZM. Independent association between nonalcoholic fatty liver disease and cardiovascular disease in the US population. Clin Gastroenterol Hepatol. 2012;10(6):646-50.
17. Eslam M, Sanyal AJ, George J. MAFLD: A Consensus-Driven Proposed Nomenclature for Metabolic Associated Fatty Liver Disease. Gastroenterology. 2020;158(7):1999-2014.e1.
18. Srivastava A, Gailer R, Tanwar S, Trembling P, Parkes J, Rodger A, et al. Prospective evaluation of a primary care referral pathway for patients with non-alcoholic fatty liver disease. J Hepatol. 2019;71(2):371-8.
19. Israelsen M, Francque S, Tsochatzis EA, Krag A. Steatotic liver disease. Lancet. 2024; 404(10464):1761-78.

Supplementary Material 2: STROBE Statement—checklist of items that should be included in reports of observational studies

	Item No.	Recommendation	Page No.
**Title and abstract**	1	(*a*) Indicate the study's design with a commonly used term in the title or the abstract	1
		(*b*) Provide in the abstract an informative and balanced summary of what was done and what was found	2-3
**Introduction**			
Background/rationale	2	Explain the scientific background and rationale for the investigation being reported	5-7
Objectives	3	State specific objectives, including any prespecified hypotheses	6-7
**Methods**			
Study design	4	Present key elements of study design early in the paper	7-11
Setting	5	Describe the setting, locations, and relevant dates, including periods of recruitment, exposure, follow-up, and data collection	7-8
Participants	6	(*a*) *Cohort study*—Give the eligibility criteria, and the sources and methods of selection of participants. Describe methods of follow-up<br>*Case-control study*—Give the eligibility criteria, and the sources and methods of case ascertainment and control selection. Give the rationale for the choice of cases and controls<br>*Cross-sectional study*—Give the eligibility criteria, and the sources and methods of selection of participants	8
		(*b*) *Cohort study*—For matched studies, give matching criteria and number of exposed and unexposed<br>*Case-control study*—For matched studies, give matching criteria and the number of controls per case	n/a
Variables	7	Clearly define all outcomes, exposures, predictors, potential confounders, and effect modifiers. Give diagnostic criteria, if applicable	9
Data sources/ measurement	8*	For each variable of interest, give sources of data and details of methods of assessment (measurement). Describe comparability of assessment methods if there is more than one group	9-10
Bias	9	Describe any efforts to address potential sources of bias	n/a
Study size	10	Explain how the study size was arrived at	n/a
Quantitative variables	11	Explain how quantitative variables were handled in the analyses. If applicable, describe which groupings were chosen and why	n/a
Statistical methods	12	(*a*) Describe all statistical methods, including those used to control for confounding	10-11
		(*b*) Describe any methods used to examine subgroups and interactions	10-11
		(*c*) Explain how missing data were addressed	11
		(*d*) *Cohort study*—If applicable, explain how loss to follow-up was addressed	n/a

		Case-control study—If applicable, explain how matching of cases and controls was addressed<br>*Cross-sectional study*—If applicable, describe analytical methods taking account of sampling strategy	
		(*e*) Describe any sensitivity analyses	n/a
Results			
Participants	13*	(a) Report numbers of individuals at each stage of study—eg numbers potentially eligible, examined for eligibility, confirmed eligible, included in the study, completing follow-up, and analysed	11
		(b) Give reasons for non-participation at each stage	11
		(c) Consider use of a flow diagram	n/a
Descriptive data	14*	(a) Give characteristics of study participants (eg demographic, clinical, social) and information on exposures and potential confounders	11-14
		(b) Indicate number of participants with missing data for each variable of interest	n/a
		(c) *Cohort study*—Summarise follow-up time (eg, average and total amount)	n/a
Outcome data	15*	*Cohort study*—Report numbers of outcome events or summary measures over time	15-18
		Case-control study—Report numbers in each exposure category, or summary measures of exposure	
		Cross-sectional study—Report numbers of outcome events or summary measures	
Main results	16	(*a*) Give unadjusted estimates and, if applicable, confounder-adjusted estimates and their precision (eg, 95% confidence interval). Make clear which confounders were adjusted for and why they were included	18-22
		(*b*) Report category boundaries when continuous variables were categorized	n/a
		(*c*) If relevant, consider translating estimates of relative risk into absolute risk for a meaningful time period	n/a
Other analyses	17	Report other analyses done—eg analyses of subgroups and interactions, and sensitivity analyses	n/a
**Key Results**			
Key results	18	Summarise key results with reference to study objectives	22-24
Limitations	19	Discuss limitations of the study, taking into account sources of potential bias or imprecision. Discuss both direction and magnitude of any potential bias	24-26
Interpretation	20	Give a cautious overall interpretation of results considering objectives, limitations, multiplicity of analyses, results from similar studies, and other relevant evidence	24-26
Generalisability	21	Discuss the generalisability (external validity) of the study results	24-27
**Other Information**			
Funding	22	Give the source of funding and the role of the funders for the present study and, if applicable, for the original study on which the present article is based	28

*Give information separately for cases and controls in case-control studies and, if applicable, for exposed and unexposed groups in cohort and cross-sectional studies.

Note: An Explanation and Elaboration article discusses each checklist item and gives methodological background and published examples of transparent reporting. The STROBE checklist is best used in conjunction with this article (freely available on the Web sites of PLoS Medicine at http://www.plosmedicine.org/, Annals of Internal Medicine at http://www.annals.org/, and Epidemiology at http://www.epidem.com/). Information on the STROBE Initiative is available at www.strobe-statement.org.

Supplementary Material 3. Table: Liver disease (viral, genetic and autoimmune) for which all BALLETS patients were tested*

Category	Disease	Blood tests carried out on all members of the cohort (to diagnose or screen for the disease)	Method by which diagnosis was made in screen positive cases
Hepatocellular diseases	Chronic viral hepatitis C	Hepatitis C virus antibody (HCV Ab)	Viral marker positive and hepatologist's opinion
	Chronic viral hepatitis B	Hepatitis B surface viral antigen (HBV surface Ag)	Viral marker positive and hepatologist's opinion
	Metal storage disease: Iron (haemochromatosis)	Transferrin levels	Genotype performed on patient if transferrin saturation >50%
	Autoimmune hepatitis	Smooth muscle antibody	Raised antibodies and ALT or AST or globulin exceeding twice the upper limit of normal. Confirmed by the hepatologist's opinion
	Metal storage disease: Copper (Wilson's disease)	Caeruloplasmin	Low levels of caeruloplasmin and hepatologist's opinion
	α-1 Antitrypsin (A1AT) deficiency	A1AT level	Phenotype testing performed if A1AT abnormal
	Alcoholic/fat-induced cirrhosis or hepatocellular cancer (HCC)	N/A	Abdominal ultrasound + exclusion of other diseases in this table and hepatologist's opinion
Intrahepatobiliary duct disease	Primary biliary cirrhosis/ cholangitis (PBC)	Antimitochondrial antibody	Anti-mitochondrial antibodies (≥1:40 titre) and hepatologist's opinion
	Primary sclerosing cholangitis	N/A	Combination raised ALP and ulcerative colitis. Confirmed by hepatologist's opinion

**We did not include the benign condition Gilbert's syndrome in any disease category.*

NB. Table taken under CC-BY-NC license from Lilford RJ, Bentham LM, Armstrong MJ, Neuberger J, Girling AJ. What is the best strategy for investigating abnormal liver function tests in primary care? Implications from a prospective study. BMJ Open. 2013; 3(6).

Supplementary Material 4. List of Diseases of the Liver and their HES code

In our ‘primary’ codes, we recorded all admissions with a record of “Disease of liver” (K70-K77). We excluded from our end-point definition viral hepatitis (B15, B16, B17), haemochromatosis (E8.3.1) and Wilson’s disease (E83.0), because all BALLETS patients were tested on entry, and have been excluded from the analysed cohort, as described above. We also planned a further, secondary analysis to improve specificity by removing codes for diseases that would most likely have arisen independently of steatosis (toxic disease of the liver [K71], unspecified biliary cirrhosis [K74.5, K75.0–K75.4], other inflammatory liver disease [K75.9], and liver disorders in other disease [K77]. In the event we did not perform this further analysis because there were less than five cases in total across these groups, and analysis would therefore be outside the terms of the data agreement (see legend to Table 3 of main document).

Table: List of Diseases of the Liver and their HES code

Code	Description	Inclusions	Exclusions
**K70**	**Alcoholic liver disease**		
K70.0	Alcoholic fatty liver		
K70.1	Alcoholic hepatitis		
K70.2	Alcoholic fibrosis and sclerosis of liver		
K70.3	Alcoholic cirrhosis of liver	Alcoholic cirrhosis Not Otherwise Specified	
K70.4	Alcoholic hepatic failure	NOS, acute, chronic, subacute, with or with rough hepatic coma	
K70.9	Alcoholic liver disease, unspecified		
**K71**	**Toxic liver disease**	**Drug induced: Idiosyncratic (unpredictable) liver disease; toxic (predictable) liver disease**	**Alcoholic liver disease; Budd-Chiari syndrome**
K71.0	Toxic liver disease with cholestasis	Cholestasis with hepatocyte injury; "Pure" cholestasis	
K71.1	Toxic liver disease with hepatic necrosis	Hepatic failure (acute)(chronic) due to drugs	
K71.2	Toxic liver disease with acute hepatitis		
K71.3	Toxic liver disease with chronic persistent hepatitis		
K71.4	Toxic liver disease with chronic lobular hepatitis		
K71.5	Toxic liver disease with chronic active hepatitis	Toxic liver disease with lupoid hepatitis	
K71.6	Toxic liver disease with hepatitis, not elsewhere classified		
K71.7	Toxic liver disease with fibrosis and cirrhosis of liver		
K71.8	Toxic liver disease with other disorders of liver	toxic liver disease with: focal nodular hyperplasia; hepatic granulomas; peliosis hepatis; veno-occlusive disease of liver	
K71.9	Toxic liver disease, unspecified		
**K72**	**Hepatic Failure, not elsewhere classified**	**hepatic coma NOS; hepatic encephalopathy NOS; acute/fulminant/malignant hepatitis NEC, with hepatic failure; liver (cell) necrosis with hepatic failure; yellow liver atrophy or dystrophy**	**Alcoholic hepatic failure; hepatic failure complicating: abortion or ectopic or molar pregnancy; pregnancy, childbirth and the pueperium; icterus of fetus and newborn; viral hepatitis; with toxic liver disease**
K72.0	Acute and subacute hepatic failure		
K72.1	Chronic hepatic failure		
K72.9	Hepatic failure, unspecified		
**K73**	**Chronic Hepatitis, not elsewhere classified**		**alcoholic/drug-induced/granulomatous NEC/reactive/viral hepatitis (chronic)**

K73.0	Chronic persistent hepatitis, not elsewhere classified		
K73.1	Chronic lobular hepatitis, not elsewhere classified		
K73.2	Chronic active hepatitis, not elsewhere classified	Lupoid hepatitis NED	
K73.8	Other chronic hepatitis, not elsewhere classified		
K73.9	Chronic hepatitis, unspecified		
**K74**	**Fibrosis and cirrhosis of liver**		**alcoholic fibrosis of liver; cardiac sclerosis of liver; alcoholic/congenital cirrhosis of liver; with toxic liver disease**
K74.0	Hepatic fibrosis		
K74.1	Hepatic sclerosis		
K74.2	Hepatic fibrosis with hepatic sclerosis		
K74.3	Primary biliary cirrhosis	Chronic nonsuppurative destructive cholangitis	
K74.4	Secondary biliary cirrhosis		
K74.5	Biliary cirrhosis, unspecified		
K74.6	Other and unspecified cirrhosis of liver	NOS/cryptogenic/macronodular/micronodular /mixed type/portal/postnecrotic cirrhosis of liver	
**K75**	**Other inflammatory liver diseases**		**Chronic hepatitis NEC; Acute or subacute hepatitis; viral hepatitis; toxic liver disease**
K75.0	Abscess of liver	NOS/Cholangitic/Heamatogenic/lymphogenic /pylephlebitic Hepatic abscess	Amoebic live abscess; cholangitis without liver disease; pylephlebitis without liver disease
K75.1	Phlebitis of portal vein	Pyelephlephitis	pyelephlebitic liver abscess
K75.2	Nonspecific reactive hepatitis		
K75.3	Granulomatous hepatitis, not elsewhere classified		
K75.4	Autoimmune hepatitis		
K75.8	Other specified inflammatory liver diseases	Nonalcoholic steatohepatitis (NASH)	
K75.9	Inflammatory liver disease, unspecified	Hepatitis NOS	
**K76**	**Other Diseases of liver**		**alcoholic liver disease; amyloid degeneration of liver; cystic disease of liver (congenital); hepatic vein thrombosis; hepatomegaly NOS; portal vein thrombosis; toxic liver disease**
K76.0	Fatty (change of) liver, not elsewhere classified	Nonalcoholic fatty liver disease (NAFLD)	nonalcoholic steatohepatitis
K76.1	Chronic passive congestion of liver	Cardiac cirrhosis (so-called)/sclerosis of liver	
K76.2	Central haemorrhagic necrosis of liver		liver necrosis with hepatic failure
K76.3	Infarction of liver		
K76.4	Peliosis hepatis	Hepatic angiomatosis	
K76.5	Hepatic veno-occlusive disease		Budd-Chiari syndrome
K76.6	Portal hypertension		
K76.7	Hepatorenal syndrome		following labour and delivery
K76.8	Other specified diseases of liver	Simple cyst of liver; Focal nodular hyperplasia of liver; Hepatoptosis	
K76.9	Liver disease, unspecified		

**K77**	**Liver disorders in diseases classified elsewhere**		
K77.0	Liver disorders in infectious and parasitic diseases classified elsewhere	cytomegaloviral/herpesviral [herpes simplex] /toxoplasma hepatitis; Hepatosplenic schistosomiasis; Portal hypertension in schistosomiasis; Syphilitic liver disease	
K77.8	Liver disorders in other diseases classified elsewhere	Hepatic granulomas in:berylliosis/sarcoidosis	

Supplementary Material 5. Figure: Analyses conducted to assess the proportional hazards assumption

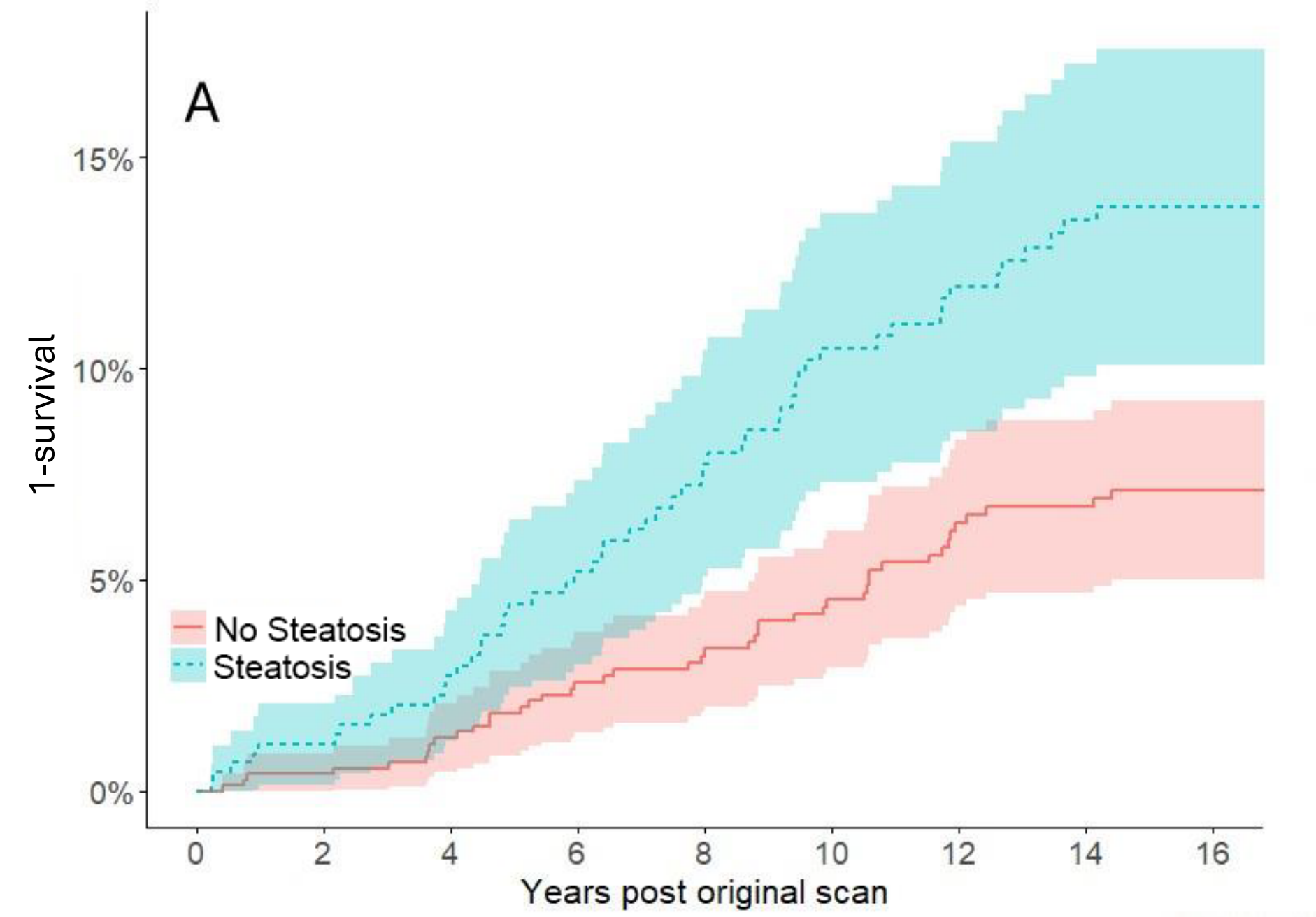

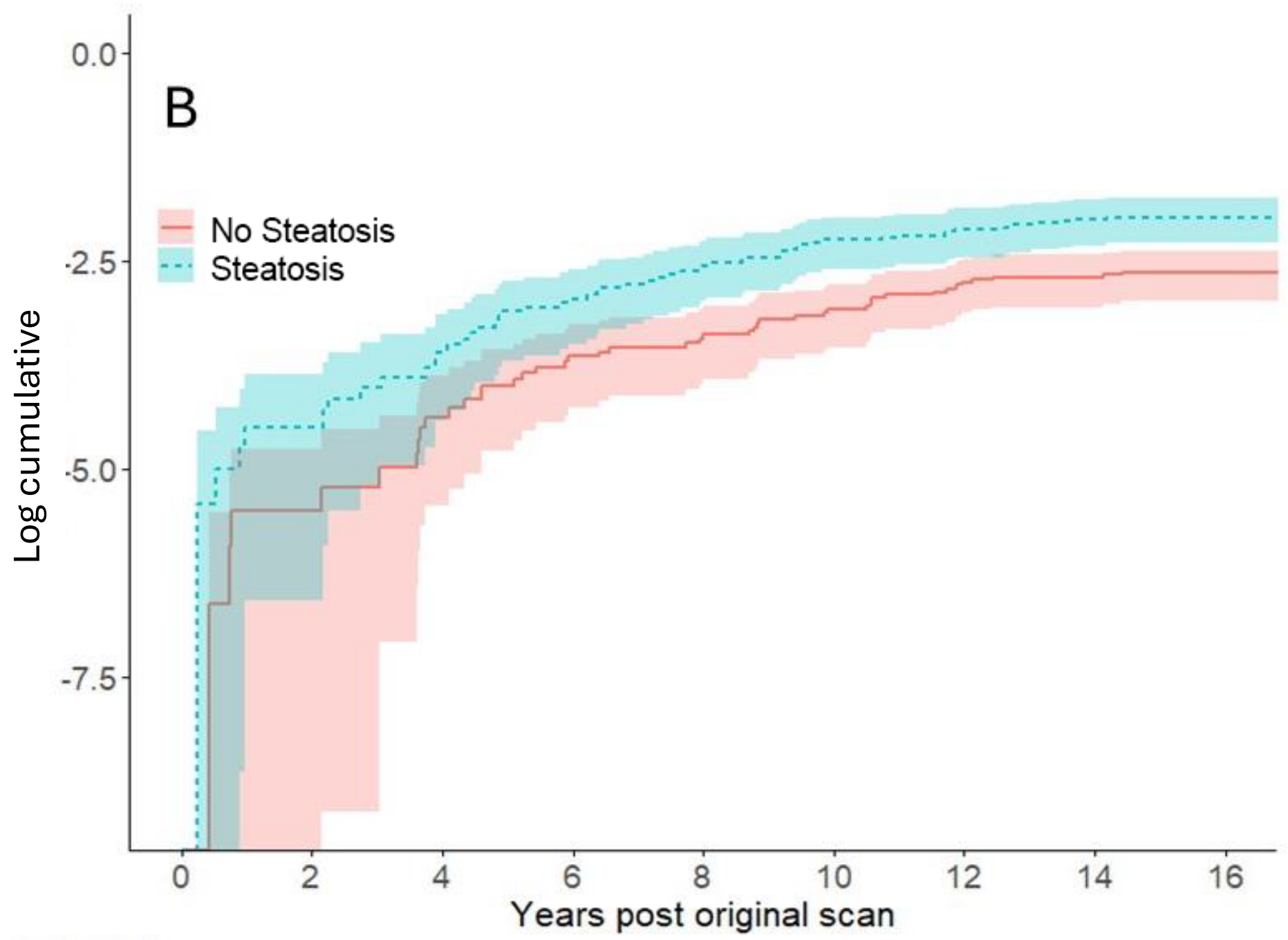

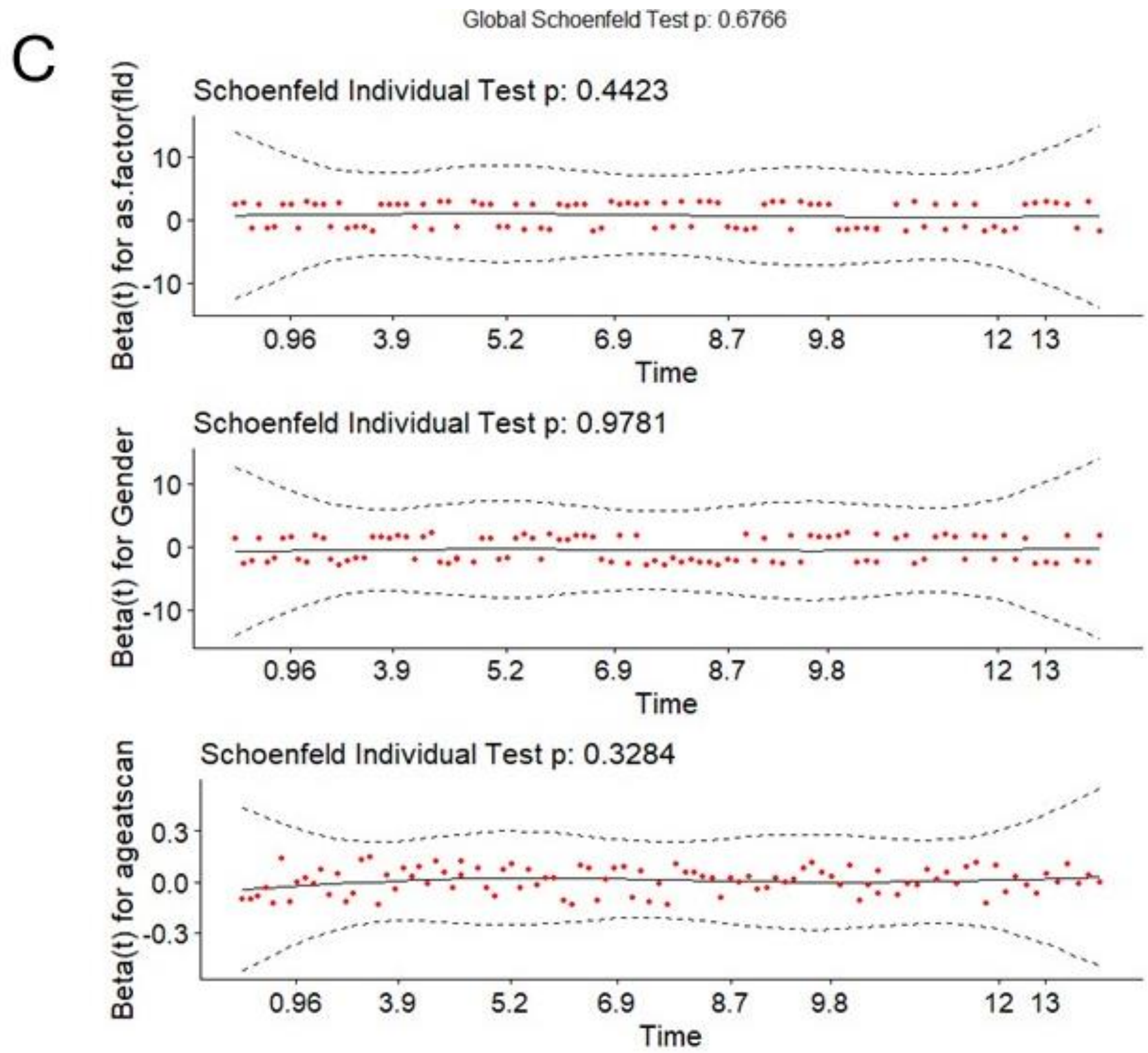

Figures above show the unadjusted primary liver outcome analyses, panel A is the cumulative hazard split by the differing severity of steatosis, panel B is the log cumulative hazard, and panel C is the Schoenfeld residuals plots.

Supplementary Material 6. Table: Individual comorbidity results

	Steatosis Status		
Demographics	**Not Present**, N = 760	**Present**, N = 455	**Total** N=1215
Cardiac arrythmias[1]	53 (7·0%)	19 (4·2%)	72 (5·9%)
Chronic pulmonary disease[1]	107 (14·1%)	47 (10·3%)	154 (12·7%)
Depression[1]	77 (10·1%)	57 (12·5%)	134 (11·0%)
Hypertension (uncomplicated)[1]	254 (33·4%)	183 (40·2%)	437 (36·0%)
Hypothyroidism[1]	46 (6·1%)	22 (4·8%)	68 (5·6%)
Other neurologic disorders[1]	55 (7·2%)	28 (6·2%)	83 (6·8%)
Renal failure[1]	81 (10·7%)	32 (7·0%)	113 (9·3%)
Rheumatoid arthritis/collagen vascular diseases[1]	40 (5·3%)	25 (5·5%)	65 (5·3%)
Solid tumor[1]	56 (7·4%)	26 (5·7%)	82 (6·7%)
Cerebrovascular disease[2]	28 (3·7%)	13 (2·9%)	41 (3·4%)
Myocardial infarction[2]	24 (3·2%)	14 (3·1%)	38 (3·1%)
Diabetes (uncomplicated)[3]	117 (15·4%)	149 (32·7%)	266 (21·9%)

[1] Based on the Elixhauser classification.

[2] Based on the Charlson Comorbidity classification.

[3] Both comorbidity classifications were identical.

Supplementary Material 7. Table: Results of the analyte performed at baseline by steatosis status, and reason original testing was done

	Steatosis Status		
Demographics	**Not Present**, N = 760	**Present**, N = 455	**Total** N=1215
ALT			
Abnormal	192 (25·3%)	214 (47·0%)	406 (33·4%)
Normal	475 (62·5%)	166 (36·5%)	641 (52·8%)
Not Tested	93 (12·2%)	75 (16·5%)	168 (13·8%)
AST			
Abnormal	111 (14·6%)	127 (27·9%)	238 (19·6%)
Normal	567 (74·6%)	290 (63·7%)	857 (70·5%)
Not Tested	82 (10·8%)	38 (8·4%)	120 (9·9%)
Bilirubin			
Abnormal	82 (10·8%)	28 (6·2%)	110 (9·1%)
Normal	666 (87·6%)	416 (91·4%)	1,082 (89·1%)
Not Tested	12 (1·6%)	11 (2·4%)	23 (1·9%)
ALP			
Abnormal	129 (17·0%)	40 (8·8%)	169 (13·9%)
Normal	625 (82·2%)	404 (88·8%)	1,029 (84·7%)
Not Tested	6 (0·8%)	11 (2·4%)	17 (1·4%)
GGT			
Abnormal	492 (64·7%)	314 (69·0%)	806 (66·3%)
Normal	189 (24·9%)	87 (19·1%)	276 (22·7%)
Not Tested	79 (10·4%)	54 (11·9%)	133 (10·9%)
Albumin			
Abnormal	54 (7·1%)	10 (2·2%)	64 (5·3%)
Normal	701 (92·2%)	438 (96·3%)	1,139 (93·7%)
Not Tested	5 (0·7%)	7 (1·5%)	12 (1·0%)
Globulin			
Abnormal	37 (4·9%)	16 (3·5%)	53 (4·4%)
Normal	549 (72·2%)	322 (70·8%)	871 (71·7%)
Not Tested	174 (22·9%)	117 (25·7%)	291 (24·0%)
T-Protein			
Abnormal	59 (7·8%)	32 (7·0%)	91 (7·5%)
Normal	529 (69·6%)	308 (67·7%)	837 (68·9%)
Not Tested	172 (22·6%)	115 (25·3%)	287 (23·6%)
Reason for test			
Abdominal discomfort	24 (3·1%)	9 (2·0%)	33 (2·7%)
Alcohol	5 (0·7%)	9 (2·0%)	14 (1·2%)
Blood Pressure	89 (11·7%)	57 (12·6%)	146 (12·0%)
Bowel Changes	11 (1·4%)	4 (0·9%)	15 (1·2%)
Bowel Disease	11 (1·4%)	1 (0·2%)	12 (1·0%)
Breathing problems	11 (1·4%)	2 (0·4%)	13 (1·1%)
CHD	24 (3·2%)	5 (1·1%)	29 (2·4%)
Check up	33 (4·3%)	18 (4·0%)	51 (4·2%)
Chest Infection, cough, Flu	10 (1·3%)	3 (0·7%)	13 (1·1%)
Chest Pain	9 (1·2%)	6 (1·3%)	15 (1·2%)
Cholesterol	34 (4·5%)	21 (4·6%)	55 (4·5%)
Diabetes	86 (11·3%)	117 (25·8%)	203 (16·7%)
Dizziness	12 (1·6%)	1 (0·2%)	13 (1·1%)
Fatigue	39 (5·1%)	15 (3·3%)	54 (4·5%)
General Malaise	6 (0·8%)	6 (1·3%)	12 (1·0%)
Indigestion, Nausea, Vomiting	15 (2·0%)	8 (1·8%)	23 (1·9%)
Medication review	39 (5·1%)	28 (6·2%)	67 (5·5%)
Mental Health	13 (1·7%)	9 (2·0%)	22 (1·8%)
Other	73 (9·6%)	40 (8·8%)	113 (9·3%)
Other Cardiac	19 (2·5%)	12 (2·6%)	31 (2·6%)
Other Pain	28 (3·7%)	9 (2·0%)	37 (3·1%)
Rash, Itch	12 (1·6%)	6 (1·3%)	18 (1·5%)
Rheumatology	19 (2·5%)	9 (2·0%)	28 (2·3%)
Thyroid	19 (2·5%)	3 (0·7%)	22 (1·8%)
Unknown	101 (13·2%)	54 (11·7%)	155 (12·6%)
Weight Loss	18 (2·4%)	3 (0·7%)	21 (1·7%)
Test Routinely done	384 (50·6%)	260 (57·3%)	644 (53·1%)

Supplementary Material 8: Figure: Frequency ICD10 Coding Among Patients with Primary Liver Outcome

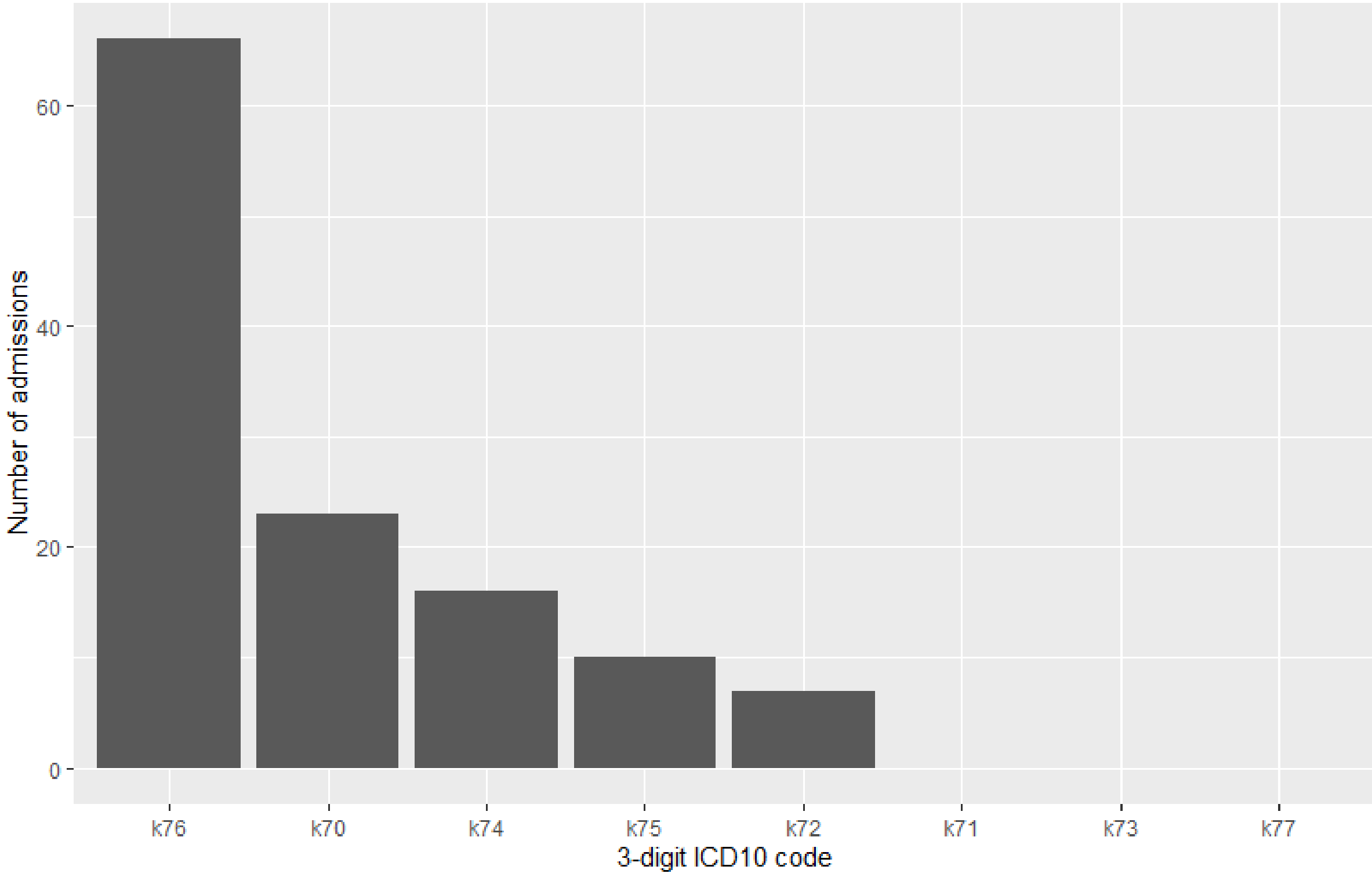

K76: Other disease of liver (including fatty change not elsewhere classified)
K70: Alcoholic liver disease
K74: Fibrosis and cirrhosis of the liver
K75: Other inflammatory liver disease
K72: Hepatic failure not elsewhere classified
K71: Toxic liver disease
K73: Chronic hepatitis not elsewhere classified
K77: Liver disorders in diseases classified elsewhere

NB. The numbers sum to more than 90 because a person could have more than one primary liver outcome ICD10 code.

Supplementary Material 9. Figure: Description of the status of patients in the cohort.

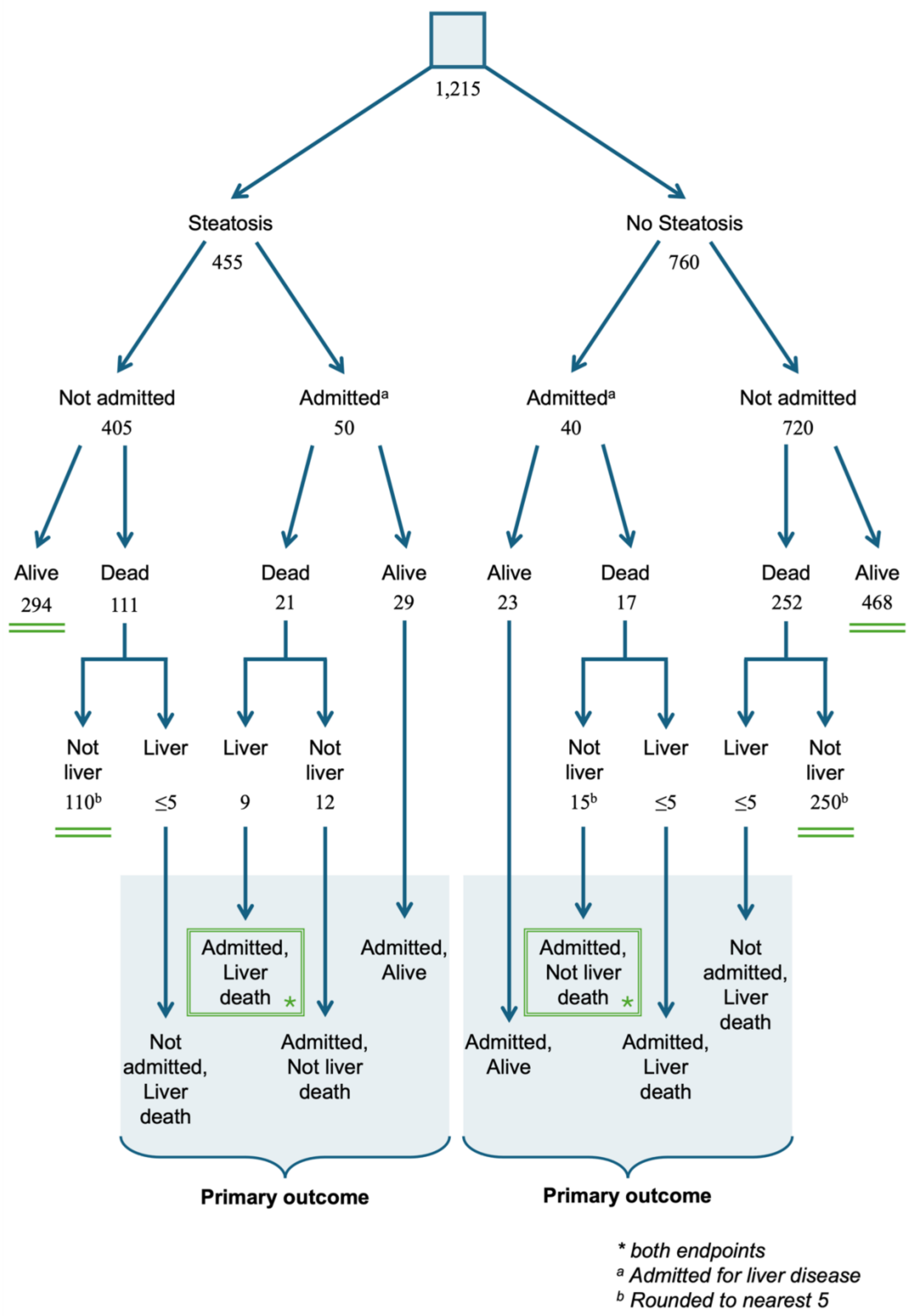

Supplementary Material 10. Secondary liver outcomes

Admissions where liver disease was a secondary reason for admission. Patients that did not have a primary liver admission may nevertheless have had an admission with liver disease recorded as a secondary reason for admission. In addition to 90 patients with an admission in the primary position, 46 patients had at least one admission with a liver diagnosis code in any position other than the primary one (liver comorbidity). Twenty seven (5.9%) and 19 ((2.5%) of these admissions were in the fatty and not fatty groups respectively.

Visit to liver outpatient department. Even if a participant was not admitted with liver disease, they may still have attended an outpatient department in relation to possible liver disease. Overall, 176 patients had at least one liver outpatient attendance, of whom 78 (44.3%) did have steatosis versus 98 (55.7%) who did not have steatosis. However, only 95 patients had an outpatient appointment with a liver consultant who were not also admitted with a (primary or secondary) diagnosis on liver disease at some time. Of these 33 (7.3%) were in the steatosis group versus 62 (8·2%) in the not steatosis group.

Hospital visits of any type. Two hundred and thirty-one people had at least one contact with a hospital for a liver condition at some point. One hundred and ten were in the steatosis group (24.2%) versus 121 (15.9%) in the not steatosis group.

Figure: Breakdown of final cohort of patients by presence of steatosis and by hospital attendance

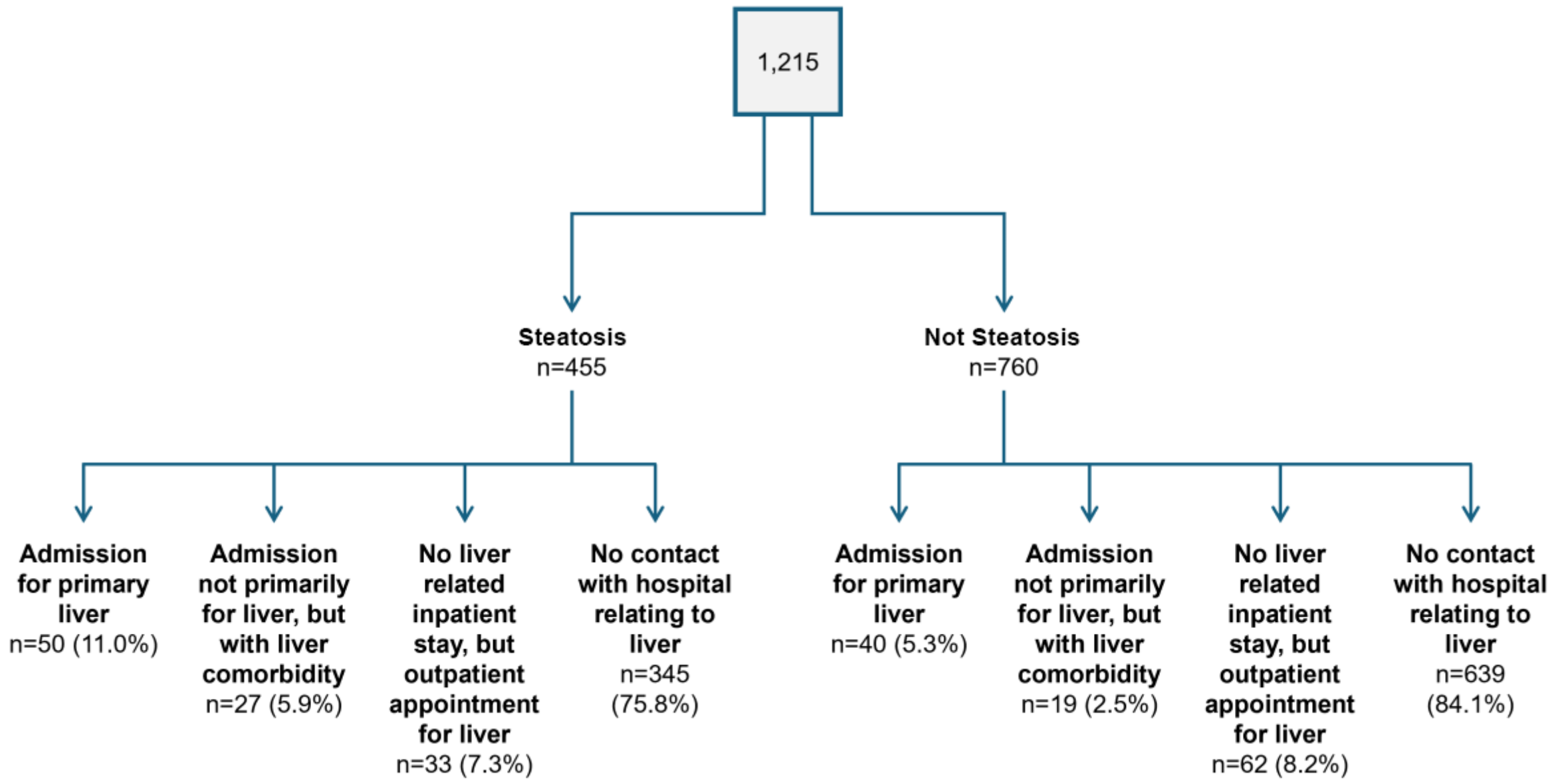

Supplementary Material 11. Figure: Graphical posterior predictive model checks for the primary outcome

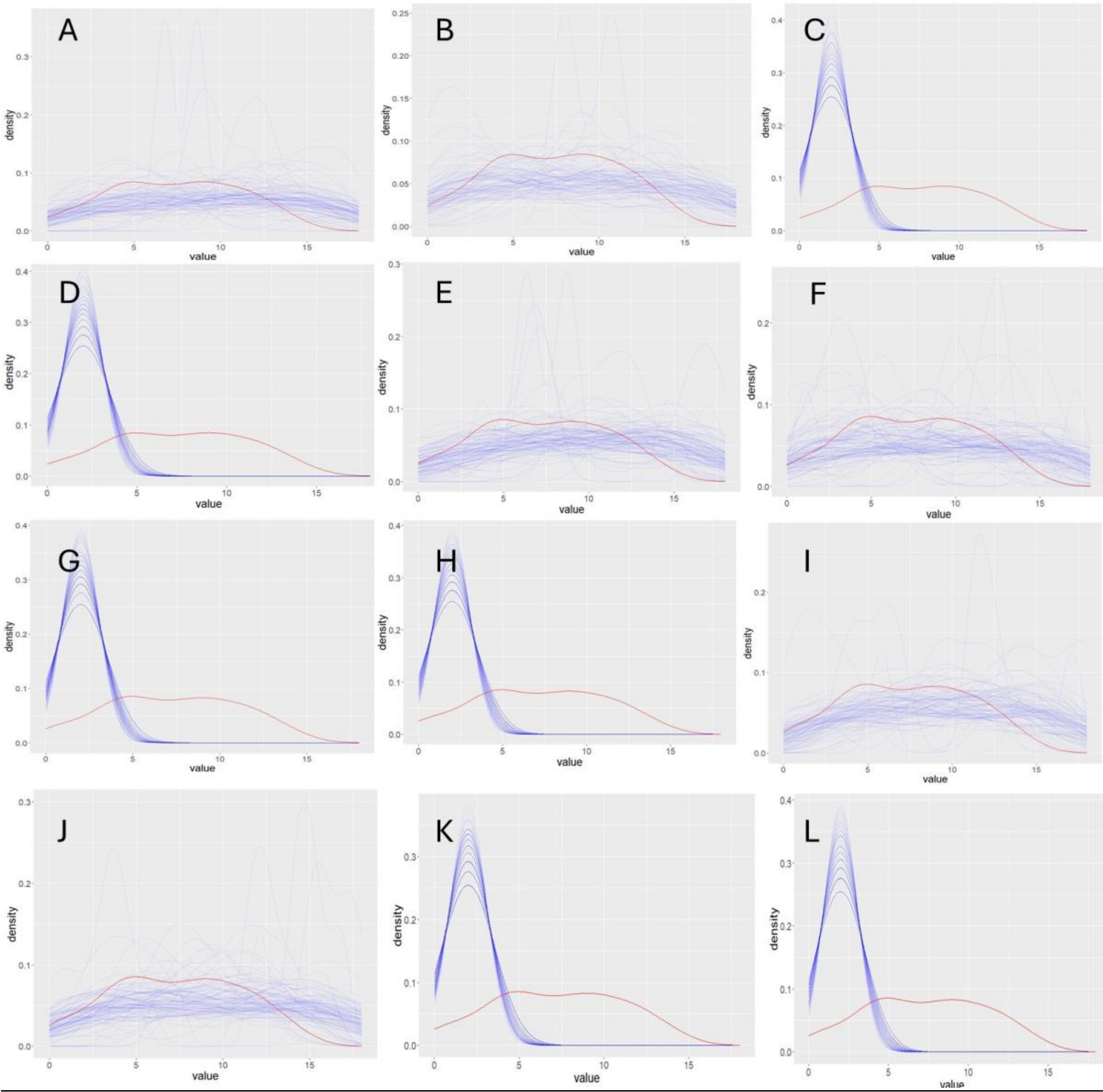

Blue lines represent sample from the posterior predictive distribution of each model, and the red line is the density of the data. Model A-L are defined in Supplementary Materials 11 and 12.

Supplementary Material 12. Table: Goodness of fit results for the different models looking at the primary outcome of admission for liver disease or death due to liver disease.

Model	Hazard	Variables	WAIC	LooIC
**A**	**Weibull**	**Standardised age, age-squared, Sex, Steatosis status, Standardised BMI, BMI-squared, Alcohol Risk**	1145.2 (94.0)	1145.2 (94.0)
B	Exponential	Standardised age, age-squared, Sex, Steatosis status, Standardised BMI, BMI-squared, Alcohol Risk	1146.١ (94.1)	1146.1 (94.1)
C	Spline- Basis 3	Standardised age, age-squared, Sex, Steatosis status, Standardised BMI, BMI-squared, Alcohol Risk	1137.7 (93.4)	1137.7 (93.4)
D	Spline – Basis 5	Standardised age, age-squared, Sex, Steatosis status, Standardised BMI, BMI-squared, Alcohol Risk	1139.8 (93.6)	1139.8 (93.6)
**E**	**Weibull**	**Standardised age, age-squared, Sex, Steatosis status**	1170.9 (94.8)	1170.9 (94.8)
F	Exponential	Standardised age, age-squared, Sex, Steatosis status	1170.6 (94.8)	1170.6 (94.8)
G	Spline- Basis 3	Standardised age, age-squared, Sex, Steatosis status	1163.3 (94.2)	1163.3 (94.2)
H	Spline – Basis 5	Standardised age, age-squared, Sex, Steatosis status	1164.8 (94.4)	1164.8 (94.4)
**I**	**Weibull**	Steatosis status	1171.4 (94.8)	1171.4 (94.8)
J	Exponential	Steatosis status	1170.7 (94.8)	1170.7 (94.8)
K	Spline- Basis 3	Steatosis status	1163.7 (94.2)	1163.7 (94.2)
L	Spline – Basis 5	Steatosis status	1165.1 (94.4)	1165.1 (94.4)

WAIC: Watanabe–Akaike information criterion.
LooIC: Leave one out information criterion.

Supplementary Material 13. Table: Hazard Ratios for all models

	HR (95%CI) Model I	HR (95%CI) Model J	HR (95%CI) Model K	HR (95%CI) Model E	HR (95%CI) Model F	HR (95%CI) Model G	HR (95%CI) Model A	HR (95%CI) Model B	HR (95%CI) Model C
FLD	2·00 (1·33, 2·94)	2·00 (1·32, 2·93)	2·01 (1·32, 2·97)	2·16 (1·38, 3·21)	2·15 (1·40, 3·17)	2·18 (1·40, 3·22)	1·97 (1·23, 3·01)	1·96 (1·23, 2·97)	1·99 (1·25, 3·02)
Standardised Age				1·14 (0·89, 1·43)	1·12 (0·87, 1·40)	1·13 (0·88, 1·41)	1·20 (0·92, 1·51)	1·17 (0·91, 1·48)	1·19 (0·92, 1·50)
Standardised Age Squared				1·05 (0·84, 1·27)	1·04 (0·84, 1·25)	1·05 (0·85, 1·26)	1·10 (0·88, 1·34)	1·09 (0·87, 1·32)	1·10 (0·89, 1·34)
Male Gender				0·65 (0·43, 0·95)	0·65 (0·42, 0·96)	0·65 (0·43, 0·93)	0·60 (0·38, 0·90)	0·60 (0·38, 0·90)	0·60 (0·39, 0·90)
Alcohol Risk (high)							1·77 (1·12, 2·67)	1·77 (1·12, 2·65)	1·78 (1·12, 2·67)
Standardised BMI							1·18 (0·89, 1·55)	1·18 (0·89, 1·55)	1·18 (0·89, 1·53)
Standardised BMI squared							0·93 (0·79, 1·04)	0·93 (0·80, 1·04)	0·93 (0·80, 1·04)

Supplementary Material 14. Overall Survival Analysis (all-cause mortality)

For a comparison with general population risk of all-cause mortality, we re-estimated the age- and sex-adjusted survival models described in the main text for the all-cause mortality endpoint. Given the negligible rate of mortality in the younger members of the cohort, we only included patients aged 65 and over at the time of the original BALLETS study in this analysis. We planned to predict the probability of all-cause mortality by age and compare to the national life tables for equivalently aged general population. In the event, few deaths among younger members of the cohort led to very imprecise comparisons.

During the follow-up period, 401 patients died, of whom 20 (5%) had a cause related to liver disease recorded on their death certificate. Of those 20, the recorded causes of death included alcoholic liver disease, cirrhosis, hepatocellular carcinoma, liver failure, multi-organ failure, sepsis and others. The Figure shows the unadjusted Kaplan Meier graphs for overall survival, from which it appears that patients with steatosis disease have better survival, although the curves converge by the end of the follow-up period.

When we examine all-cause mortality within the BALLETS cohort, steatosis appears to be 'protective'. That said, the number of deaths is small, and the curves converge over time.

Figure. Unadjusted Kaplan Meier for overall survival

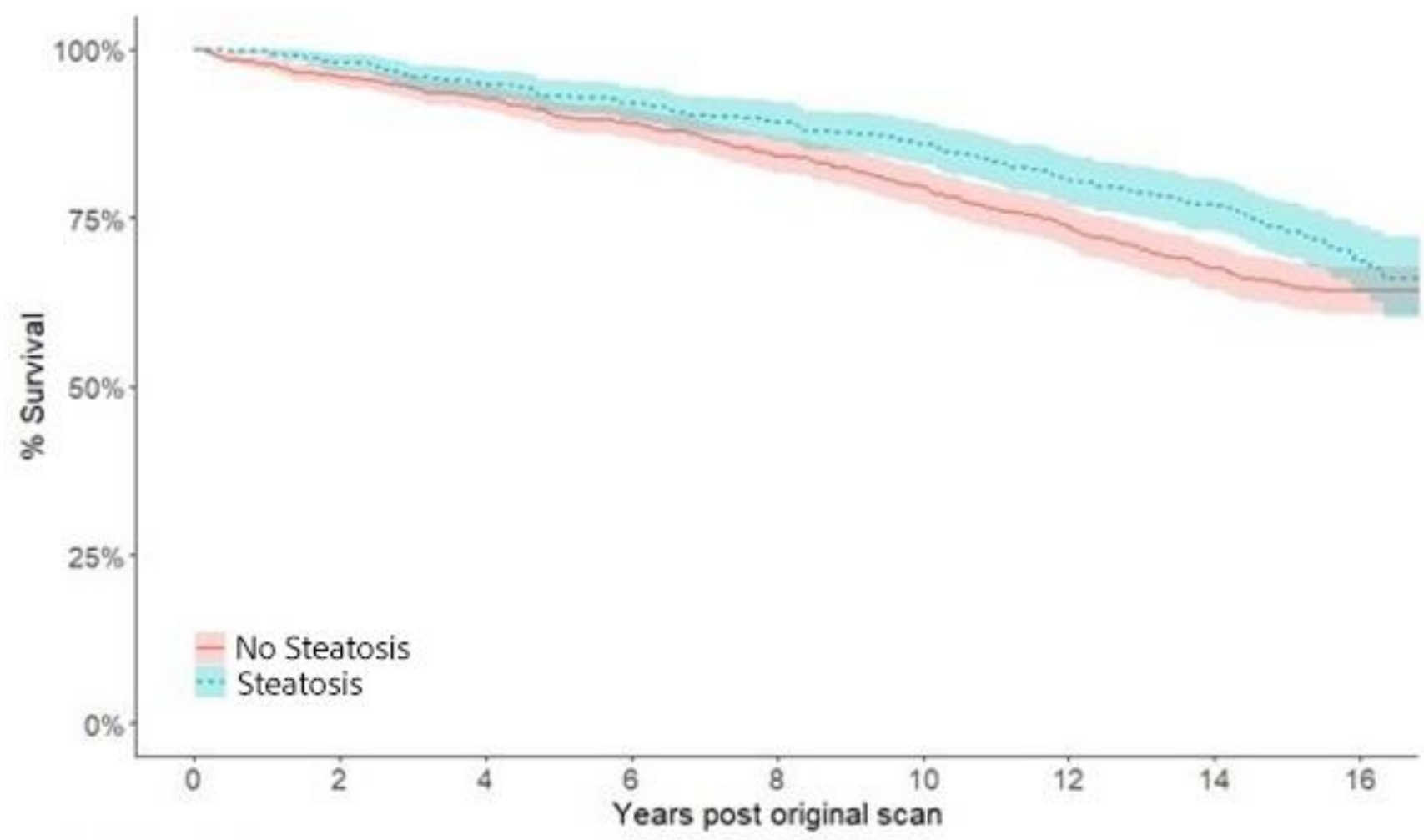

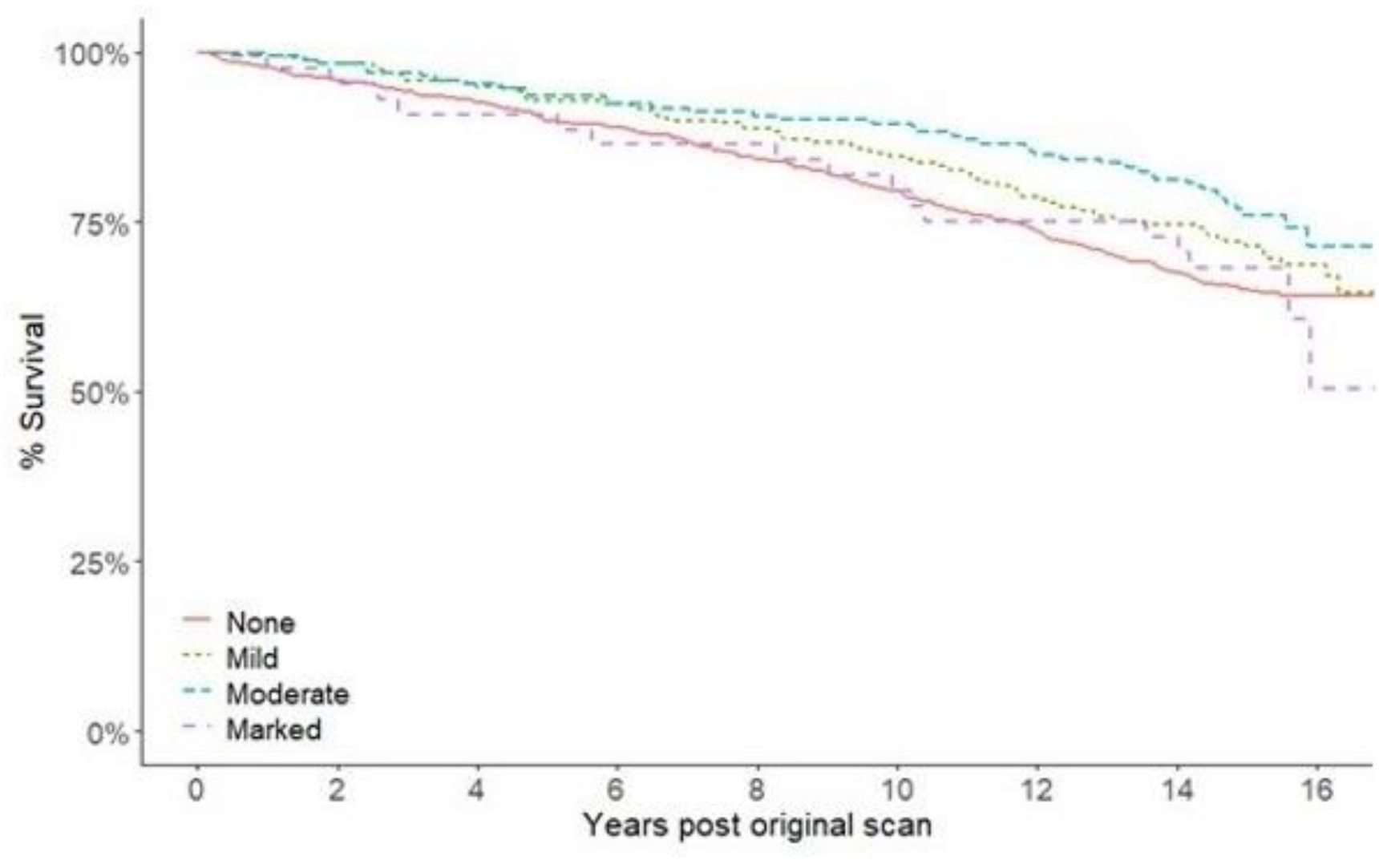

Top panel combines all degrees of steatosis, while the bottom panel decomposes steatosis by 'severity'.
