# Supplementary material for: Long-term outcomes of ultrasound-detected hepatic steatosis in primary care: a record linkage study
Source: BMJ Open. 2026 Nov 6;16(11):e124118. doi: 10.1136/bmjopen-2026-124118 (PMC13756320; doi:10.1136/bmjopen-2026-124118)
Supplement: Supplementary file 2 [file bmjopen-2026-124118.reviewer_comments.pdf]

PEER REVIEW HISTORY

BMJ Open publishes all reviews undertaken for accepted manuscripts. Reviewers are asked to complete a checklist review form and are provided with free text boxes to elaborate on their assessment. These free text comments are reproduced below.

ARTICLE DETAILS

Title (Provisional)

Long-Term Outcomes of Ultrasound-Detected Hepatic Steatosis in Primary Care - a Record Linkage Study

Authors

Lilford, Richard J; Evison, Felicity; Reeves, Katharine; Skrybant, Magdalena; Chilton, Peter J; Hofer, Timothy P.; Watson, Samuel I

VERSION 1 - REVIEW

**Reviewer**	**1**
**Name**	**Jarvis, Helen**
**Affiliation**	**Newcastle University, Institute of Health & Society**
**Date**	**16-Aug-2026**
**COI**	**None**

I am unable to comment on the detail of the appropriateness of the statistical analysis as I am not a statistician/epidemiologist and this paper requires this review from elsewhere.

Overall I think this is interesting research and of some use to guide primary care clinical practice is this area but that the framing of the discussion and conclusions needs some work before publication.

Minor points/corrections in manuscript:

Introduction:

P5 /line 13/14 -

'A recent Lancet 'Seminar' article recommends the term steatosis to describe this finding, rather than Non-Alcoholic Fatty Liver Disease (NAFLD).(4) '

The recommendation is to use the term 'steatosis of the liver' rather than 'fatty liver' to describe the finding on ultrasound. At this stage you would not use the term NAFLD/MASLD or indeed another cause of steatosis e.g ALD/MetALD as these are clinical disease

diagnoses and not findings on a scan which can only describe the radiological findings and not the cause of the condition.

P5/line 28/38

'It is important therefore to provide scientific evidence regarding the magnitude of the risk of progression since the recommendation has been made that all people with ultrasound-detected steatosis identified in primary care should be investigated by means of serial blood tests and scans of the liver,(7) and that the general population should be screened for the condition. '

Reference 7 given is to a systematic review of the global epidemiology of MASLD for 2023 and makes no mention of the recommendation that all people with US steatosis should be investigated with 'serial blood tests and scans' - ? wrong reference - ? should be using latest EASL/EASD/EASO 2024 joint MASLD guidelines?

There is no recommendation that I know of tha the 'general population should be screening for the condition' . Not clear what 'the condition' refers to but if steatosis on ultrasound then I don't know of any guidelines recommending this in the 'general population' or even in high risk groups for that matter only that if have abnormal liver blood tests it is part of the general work up but this is not 'screening'. Suggest remove this part of the sentence.

P6/line 3 - ? should be 'causes' rather than 'case'

P6/line 18 - refers to primary biliary cirrhosis - should be 'cholangitis' not 'cirrhosis'

P6/line 40 - 'family practitioner' -not a commonly used term in UK practice. Suggest use 'Primary care' throughout rather than GP/family practitioner and refer to a 'Primary Care Practitioner'

P7/line 9

'Our results will be most directly referable to such a patient, but also provide: 1. An upper limit for the probability of serious liver disease for a person with an ultrasound diagnosis, but no abnormal liver test result.'

Not sure you can say this as the study is not looking at people with normal LFTs and this is just an assumption? Suggest remove

P7 38/39 - again suggest use 'primary care' throughout rather than GP

P9 45-53

'Although our primary diagnosis concerned admissions where liver disease was primary, we also conducted a 'secondary' analysis where we examined associations between steatosis and 1) admissions where liver disease was a secondary cause and 2) outpatient department attendance under the care of a liver consultant. Statistical analyses were not carried out in these groups.'

Not sure this makes sense - state that you conducted a secondary analysis but then state that not analyses were carried out in these groups?

P11 35-40
Mentions mild/moderate/severe steatosis and then mentions grades of steatosis later - can you make clearer how these were defined e.g. % fat /other radiological parameters?? (sorry if I missed this elsewhere)

P12 26-28
You mention that the steatosis cohort were more likely to have had the initial blood testing as part of routine checks e.g. diabetic checks - interesting point but not picked up further in the discussion. Could this have any implications e.g abnormal tests mean something different in pre-selected populations who are attending anyway for related metabolic co-morbidity, they are more likely to have steatosis and more likely to have liver related outcomes??

Results section - no comments from me on this but I cannot fully assess the appropriateness and rigour of the statistical methods as this is not my area of expertise.

Major point to address:

Discussion:

I feel the discussion is too long and oddly worded for a quantitative analysis with lots of narrative/opinion and presenting differing phiisopical view points on how the results should be interpreted.

I would suggest this is re-structured into summary of main findings, comparison with other literature, strengths and limitations and implications for practice/policy/further research before conclustions .
At present the list of points 1-9 appearing at the end of the discussion before the conclusions seems slightly out of place -most of this would sit better in either strenghts/limitations/justification for study design or implications for practice
I would strongly advise further work on the discussion and would like to see a revised version of this prior to considering this at publication standard.

Perhaps you have been advised/made a conscious decision to structure this in this way? In which case I would still make the following points:

1. *'In short, our findings are consistent with a recent Lancet (4) consensus guideline that abnormal analytes on the LFT panel should prompt an upper-right quadrant scan, and that if this is positive for steatosis, should in turn provoke a fibrosis '*

This summarises the section before where you present arguments re how to use the main result (that presence of steatosis is related to increased rate of liver outcomes) to guide clinical practice. I agree that it may be useful as an 'additional prompt' of severity in these patients with abnormal LFTs but the guidelines on this are already pretty clear that *anyone*

with an abnormal LFT should undergo a liver screen and if negative/no cause identified should then do on to have some sort of fibrosis assessment (with most national and international guidelines advising this is a 2 step process using Fib4 followed by either ELF/Fibroscan). The 'prompting ' of a RUQ scan /US is just part of this work-up to r/o other causes and in itself being 'positive' does not provoke the fibrosis assessment. Even if the scan was normal and there were abnormal LFTs with no specific cause found with clinically either RF for MASLD/ALD or both then fibrosis testing would be carried out. The guidelines you refer to have 'steatosis on imaging' and 'abnromal LFTs' as 2 separate routes into fibrosis testing not needing both.

2. Using terms like 'influencing care at the margins' and 'toggle a decision' and 'our design is based on our philosophical purpose' is not very useful to clinicians in guiding practice - ? sounds a bit AI generated - consider changing
3. P23 43/44 - *Our findings provide somewhat less guidance about how actively to look for steatosis disease.*
   Need to re-phrase - our results cannot directly provide clinical guidance on the question of whether to proactively look for SLD in populations or similar.
   I would not use the term 'steatosis disease' - preferred terms are steatotic liver disease (SLD) or liver steatosis (not a disease - just presence of fat on scan)
4. P24 - as above I would advise restructuring these points 1-9 into sub-headings that are more familiar to readers of scienfiic paper discussions as above

Specifically point 3; '*From a purely scientific point of view, patients with an abnormal LFT are a good control group since few patients would be referred for a liver scan if they had normal LFTs. That is to say, they most directly inform the decision on whether to order a scan for steatosis when an LFT is abnormal.'*
This point is made without any evidence/reference? In primary care I would say it is very common to refer for an abdominal US without having abnormal LFTs - it may not be a 'scan for steatosis' but rather to Ix abdominal pain ? GS/unknown cause, look for AAA, Ix for loin pain ? kiney pathology etc etc. So there are lots of incidental abnormal US showing steatosis in those with normal LFTs so not sure your assumption that 'patients with an abnormal LFT are a good control group'!!

Conclusion:

In conclusion, steatosis is not a meaningless observation and approximately doubles the risk (from ~7% to ~14%) of a liver outcome of sufficient severity to be the primary cause of admission (or death).

Make clear that this statement refers to the population 'with abnormal LFTs'

Reviewer **2**

Name **Ryan Baumann, Mary**

Affiliation **University of Wisconsin-Madison School of Medicine and Public Health, Population Health Sciences; Biostatistics and Medical Informatics**

Date 18-Aug-2026

COI None

This study links patient records to data from the BALLETS study to understand how long-term outcomes for those with steatosis detected via ultrasound scans differ from those who underwent the same scans but no steatosis was found/detected. The primary outcome is defined as a composite of 1) hospital admission with a diagnosis of liver disease, and 2) death with liver disease as a main or contributing cause. While frequency tables are broken down by combinations of these two outcomes (e.g., alive & no hospitalization; alive & at least one hospitalization; died of liver disease & no hospitalization; died of liver disease & at least one hospitalization; died of other causes & no hospitalization; died of other causes & at least one hospitalization), the main analysis was a Bayesian survival analysis using the binary composite outcome.

As the statistical reviewer, I will focus on the analysis and statistical aspects of this manuscript. Overall I find that while the premise of the analyses is sound, several points of clarification are required in the reporting and justification of methods and results. Further, I believe a significant overhaul of the discussion section is required. I outline my comments below in order of where they appear in the manuscript. All page numbers refer to manuscript page numbers located in the bottom left corner.

1. Page 8, “Outcomes” section: The authors could be clearer here that the primary outcome is the binary composite. As currently written, it could be interpreted as two co-primary outcomes on which separate survival analyses have been conducted. On a second read-through, this section reads more clearly, but I think it helps to be very explicit to avoid confusion, especially since unadjusted breakdowns of the composite categories are also reported.

2. Page 9, “Statistical Methods” section:
    a. Both in this section and in the pre-registered protocol, it’s stated that models were covariate-adjusted using: “standardised age and its square, sex, BMI and alcohol usage above the contemporary recommended units”. However, model results in Table 3 use standardized BMI and its square. The methods section should be updated to reflect this.

    b. Further, the results section includes both “age and sex only” adjusted models and “fully” adjusted models, though these separate model adjustments are not outlined in the methods section. Please 1) specify these separate models will be fit, and 2) provide justification or motivation for fitting these two types of adjusted models (i.e., what separate goals do they seek to achieve, or what are the model

comparison goals?).

   c. In addition, the authors state that they use “weakly informative prior distributions”, but do not specify what those priors are. Inspection of the pre-registered protocol indicates these were likely N(0,52), which sets a prior 95% credible interval on hazard ratios of [5.5×10^{-5}, 1.8×10^{4}]. These details should be included in the current submission.

   d. Moreover, I’d appreciate a little more justification of the priors. I appreciate the motivations for using weakly informative priors, but it does seem that hazard ratios of [5.5×10^{-5}, 1.8×10^{4}] is extremely wide and could be further narrowed without losing the “weakly informative” designation.

3. Page 12, Table 1:
   a. I’m unclear with whether the data actually captured sex assigned at birth. It seems that until relatively recently sex and gender in medical data collection forms were not explicitly and formally separated, leaving some ambiguity for patients (e.g., forms stated “sex” which many probably interpreted as sex assigned at birth but some individuals could take to mean or choose to interpret as gender). However, I’m unfamiliar with the specific data collection practices in the UK, where this study was conducted, or with the BALLETS study specifically.

   b. Please clarify over what recall period is for the number of alcohol units consumed.

4. Page 18, lines 8-16, “Results” section: *“Having a BMI over 30 only slightly increased the probability of the liver outcome, although alcohol intake was more predictive.”* I’m unclear what the authors mean by this. I would think that the authors are referring to Table 3 which shows hazard ratios, but a binary variable for BMI >30 is not used in this table. BMI >30 is referred to in Table 4 where the authors show posterior predicted probability of having a liver outcome at 5-, 10-, and 15-years post-baseline, but these results would not show comparative increase in predictability for alcohol intake. Further, Table 3 does not show that increased alcohol intake was more predictive than BMI, but shows that its 95% credible interval rules out a hazard=1 for having a liver outcome. At a minimum, these two statements should be separated and should reference their respective results tables.

5. Page 20, Table 4:
   a. I believe this table has a typo, as you cannot calculate cumulative incidence for a “no primary outcome” group. I think the authors

mean for these groups to be “no steatosis” vs “steatosis”.

b. I’m unclear precisely what the authors mean by the last row of this table that reference BMI >30. Are they looking at posterior predictive probabilities for the subgroup of patients with BMI >30? Have they replaced the BMI and squared-BMI covariates with a binary indicator variable for BMI >30?

6. “Discussion” section:
   a. *“The probability of a liver end-point over ~15 years of follow-up is increased by about seven percentage points, adjusted for age and sex, decreasing to about six percentage points when also adjusted for obesity and alcohol intake. This is likely to reffect the effect of baseline obesity and alcohol on the explanatory variable (steatosis) and the subsequent effects of ongoing excess calorie and alcohol intake.”* It seems that the authors are implying that death due to increased BMI or alcohol intake act as competing risks for liver outcomes. However, on page 24 point 6 the authors explicitly state that they do not believe competing risks is an issue for this analysis. Please clarify both of these points.

   b. The discussion regarding what should be done when steatosis is found on a scan is somewhat complicated by the fact that the present analyses do not take into account what kinds of care patients received after steatosis was/was not found. It is possible that no preventative care was administered to those where steatosis was found, or that all patients with steatosis received as much intervening care as possible.

   c. Page 22, lines 33-41: The statement that the findings of this manuscript are consistent with recent Lancet consensus guidelines conflates the question being discussed up until this point (“what should we do when we find steatosis?”) with their second proposed question (“should we actively look for [steatosis]?”). This statement should be moved to the latter part of the section where they discuss the appropriate question it references.

   d. To this point, it seems that the question “should we actively look for [steatosis]?” has already been answered by consensus guidelines and thus discussion on this point is moot.

   e. Pages 23-25: I am unclear what purpose the numbered points attempt to serve, as they appear to be a random collection of leftover thoughts. Without contextualization, I take issue with several of them, particularly point 1 (justification and context needed for inclusion of this new outcome, particularly if to be included in the primary

composite), point 2 (unclear if risk of steatosis also varies based on different global populations), point 5 (unclear the point the authors are attempting to make), and point 6 (discussed in my comment 6a) . If the authors wish to integrate these into the discussion they need to be appropriately synthesized and contextualized.

VERSION 1 - AUTHOR RESPONSE

Reviewer 1

#	Reviewer Comment	Authors' Response
**INTRODUCTION**		
1	P5, lines 13-14 – The recommendation is to use the term 'steatosis of the liver' rather than 'fatty liver' to describe the finding on ultrasound. At this stage you would not use the term NAFLD/MASLD... as these are clinical disease diagnoses and not findings on a scan.	Change made, thank you
2a	P5, lines 28-38 - Reference 7 given is to a systematic review of the global epidemiology of MASLD for 2023 and makes no mention of the recommendation that all people with US steatosis should be investigated with 'serial blood tests and scans'? Wrong reference? Should be using latest EASL/EASD/EASO 2024 joint MASLD guidelines?	Thank you, we have updated the reference to the one suggested.
2b	P5, lines 28-38 - There is no recommendation that I know of that the 'general population should be screening for the condition' . Not clear what 'the condition' refers to but if steatosis on ultrasound then I don't know of any guidelines recommending this in the 'general population' or even in high risk groups for that matter only that if have abnormal liver blood tests it is part of the	We agree and have removed the phrase relating to population screening.

	general work up but this is not 'screening'. Suggest remove this part of the sentence.	
3	P6, line 3 – should be 'causes' rather than 'case'	Correction made, thank you
4	P6, line 18 – should be 'cholangitis' not 'cirrhosis'	Correction made, thank you
5	P6, line 40 – 'family practitioner' - not a commonly used term in UK practice. Suggest use 'Primary care' throughout rather than GP/family practitioner and refer to a 'Primary Care Practitioner'	We have gone through and changed to primary care / practitioner throughout, thank you
6	P7, line 9 - 'Our results will be most directly referable to such a patient, but also provide: 1. An upper limit for the probability of serious liver disease for a person with an ultrasound diagnosis, but no abnormal liver test result.'<br>Not sure you can say this as the study is not looking at people with normal LFTs and this is just an assumption? Suggest remove.	We would like to retain this point. We think the logic holds because, as we now say, "on the assumption that people with steatosis and abnormal liver enzymes have a higher risk of progression than those with steatosis but normal enzymes."
7	P7 lines 38-39 - again suggest use 'primary care' throughout rather than GP.	Change made, thank you
8	P9, lines 45-53 - 'Although our primary diagnosis concerned admissions where liver disease was primary, we also conducted a 'secondary' analysis where we examined associations between steatosis and 1) admissions where liver disease was a secondary cause and 2) outpatient department attendance under the care of a liver consultant. Statistical analyses were not carried out in these groups.'<br>Not sure this makes sense - state that you conducted a	This is an interesting comment. Here we want to show that we recognise that alternative outcome to admission with a primary diagnosis of liver disease would have been admissions that included a secondary diagnosis or cases seen on outpatient department. Since the underlying hypothesised causal mechanism is the same, and

	secondary analysis but then state that not analyses were carried out in these groups?	thus to avoid p-hacking', we did not do a statistical hypothesis test but make the data available and the point estimate explicit. We believe that our approach is in line with American Society of Statistician recommendations [Wasserstein RL & Lazar NA. The ASA's Statement on p-Values: Context, Process, and Purpose. Am Stat. 2016; 70(2)]<br><br>To make this clear we now add:<br>'The purpose of this secondary analysis was to examine for consistency between our selected outcome (admissions with a primary diagnosis) and alternative outcomes that might have been chosen to examine the same underlying causal pathway (admissions including secondary diagnoses and cases seen only in the out-patient department).'
9	P11, lines 35-40 - Mentions mild/moderate/severe steatosis and then mentions grades of steatosis later - can you make clearer	Added text to clarify that these were categorised by the sonographer on the basis of liver texture.

	how these were defined e.g. % fat /other radiological parameters?? (sorry if I missed this elsewhere)	
10	P12, lines 26-28 - You mention that the steatosis cohort were more likely to have had the initial blood testing as part of routine checks e.g. diabetic checks - interesting point but not picked up further in the discussion. Could this have any implications e.g abnormal tests mean something different in pre-selected populations who are attending anyway for related metabolic co-morbidity, they are more likely to have steatosis and more likely to have liver related outcomes??	Thank you for this suggestion. We now include at the end of the first paragraph of the discussion;<br>'The steatosis cohort were more likely to have had initial blood testing as part of routine checks for conditions like diabetes rather than presenting with symptoms. This is consistent with the theory that steatosis is a feature of an underlying metabolic syndrome.
11	I feel the discussion is too long and oddly worded for a quantitative analysis with lots of narrative/opinion and presenting differing phiisopical view points on how the results should be interpreted. I would suggest this is re-structured into summary of main findings, comparison with other literature, strengths and limitations and implications for practice/policy/further research before conclustions.	We have followed the suggested headings, and we have made it less speculative. Thank you.
12	At present the list of points 1-9 appearing at the end of the discussion before the conclusions seems slightly out of place - most of this would sit better in either strenghts/limitations/justification for study design or implications for practice	We agree and have followed this suggestion.

13	I would strongly advise further work on the discussion and would like to see a revised version of this prior to considering this at publication standard.	We have focussed the discussion on issues rising more directly from our data and hope that you approve of the result.
14	Perhaps you have been advised/made a conscious decision to structure this in this way?	We have taken your advice for this new version!
15	… In which case I would still make the following points:<br>'In short, our findings are consistent with a recent Lancet (4) consensus guideline that abnormal analytes on the LFT panel should prompt an upper-right quadrant scan, and that if this is positive for steatosis, should in turn provoke a fibrosis '<br>This summarises the section before where you present arguments re how to use the main result (that presence of steatosis is related to increased rate of liver outcomes) to guide clinical practice. I agree that it may be useful as an 'additional prompt' of severity in these patients with abnormal LFTs but the guidelines on this are already pretty clear that anyone with an abnormal LFT should undergo a liver screen and if negative/no cause identified should then do on to have some sort of fibrosis assessment (with most national and international guidelines advising this is a 2 step process using Fib4 followed by either ELF/Fibroscan). The 'prompting ' of a RUQ scan /US is just part of this work-up to r/o other causes and in itself being 'positive' does not provoke the fibrosis assessment. Even if the scan was normal and there were abnormal LFTs with no specific cause found with	We have retained the point about fibrosis testing for steatosis and also noted that the guideline recommends a fibrosis test for abnormal LFTs net of steatosis when there is no other explanation.

	clinically either RF for MASLD/ALD or both then fibrosis testing would be carried out. The guidelines you refer to have 'steatosis on imaging' and 'abnromal LFTs' as 2 separate routes into fibrosis testing not needing both.	
	Using terms like 'influencing care at the margins' and 'toggle a decision' and 'our design is based on our philosophical purpose' is not very useful to clinicians in guiding practice - ? sounds a bit AI generated - consider changing	The phrases were not AI generated but we have amended the language
	P23 43/44 - Our findings provide somewhat less guidance about how actively to look for steatosis disease.<br>Need to re-phrase - our results cannot directly provide clinical guidance on the question of whether to proactively look for SLD in populations or similar.	We have removed this section as it goes beyond our findings and may serve no useful purpose.
	I would not use the term 'steatosis disease' - preferred terms are steatotic liver disease (SLD) or liver steatosis (not a disease - just presence of fat on scan)	Changes made, thank you
	P24 - as above I would advise restructuring these points 1-9 into sub-headings that are more familiar to readers of scienfiic paper discussions as above	We agree and have done this.
	Specifically point 3; 'From a purely scientific point of view, patients with an abnormal LFT are a good control group since few patients would be referred for a liver scan if they had normal LFTs. That is to say, they most directly inform the decision on whether to order a scan for steatosis when an LFT is abnormal.'<br>This point is made without any evidence/reference? In primary	We have removed this point.

	care I would say it is very common to refer for an abdominal US without having abnormal LFTs - it may not be a ‘scan for steatosis’ but rather to Ix abdominal pain ? GS/unknown cause, look for AAA, Ix for loin pain ? kiney pathology etc etc. So there are lots of incidental abnormal US showing steatosis in those with normal LFTs so not sure your assumption that ‘patients with an abnormal LFT are a good control group’!!	
**CONCLUSION**		
	In conclusion, steatosis is not a meaningless observation and approximately doubles the risk (from ~7% to ~14%) of a liver outcome of sufficient severity to be the primary cause of admission (or death).<br>Make clear that this statement refers to the population ‘with abnormal LFTs’	Thank you, we have adopted your suggestion.

Reviewer 2

#	Reviewer Comment	Authors’ Response
**INTRODUCTION**		
0	Overall, I find that while the premise of the analyses is sound, several points of clarification are required in the reporting and justification of methods and results. Further, I believe a significant overhaul of the discussion section is required…	Your point is well taken and we have re-written the discussion section (as both reviewers have suggested).
1	Page 8, “Outcomes” section: The authors could be clearer here that the primary outcome is the binary composite. As currently written, it could be interpreted as two co-primary outcomes on which separate survival analyses have been conducted. On a	Thank you. We now make this clearer as follows:<br>Our primary outcome is the binary composite of 1) hospital admission

	second read-through, this section reads more clearly, but I think it helps to be very explicit to avoid confusion, especially since unadjusted breakdowns of the composite categories are also reported.	with a primary diagnosis of liver disease………..and/or death where liver disease…………….'
2a	Page 9, "Statistical Methods" section: Both in this section and in the pre-registered protocol, it's stated that models were covariate-adjusted using: "standardised age and its square, sex, BMI and alcohol usage above the contemporary recommended units". However, model results in Table 3 use standardized BMI and its square. The methods section should be updated to reflect this.	Thank you for drawing our attention to this inconsistency. We now write: 'standardised BMI and its square….'
2b	Page 9, "Statistical Methods" section: Further, the results section includes both "age and sex only" adjusted models and "fully" adjusted models, though these separate model adjustments are not outlined in the methods section. Please 1) specify these separate models will be fit, and 2) provide justification or motivation for fitting these two types of adjusted models (i.e., what separate goals do they seek to achieve, or what are the model comparison goals?).	Thank you. We now explicate the goals as follows: "We fit unadjusted models, models adjusted for age and sex alone, and then, separately, fit a fully adjusted model so as to adjust for potential confounders while separately 'exploring' variables of clinical interest."
2c	Page 9, "Statistical Methods" section: In addition, the authors state that they use "weakly informative prior distributions", but do not specify what those priors are. Inspection of the pre-registered protocol indicates these were likely N(0,52), which sets a prior	We now specify this prior in the main text: "Specifically, we used a $N(0, 5^2)$ prior for log-hazard ratio intervals."

	95% credible interval on hazard ratios of [5.5×10−5, 1.8×104]. These details should be included in the current submission.	
2d	Page 9, "Statistical Methods" section: Moreover, I'd appreciate a little more justification of the priors. I appreciate the motivations for using weakly informative priors, but it does seem that hazard ratios of [5.5×10−5, 1.8×104] is extremely wide and could be further narrowed without losing the "weakly informative" designation.	We opted for a wide prior distribution here, which is almost "flat" but provides some regularisation. Other weakly informative prior distributions would provide the same effect. We have made an amendment to the text:<br>"… among the whole cohort, we also report predicted probabilities among the subgroup with BMI >30."
3a	Page 12, Table 1: I'm unclear with whether the data actually captured sex assigned at birth. It seems that until relatively recently sex and gender in medical data collection forms were not explicitly and formally separated, leaving some ambiguity for patients (e.g., forms stated "sex" which many probably interpreted as sex assigned at birth but some individuals could take to mean or choose to interpret as gender). However, I'm unfamiliar with the specific data collection practices in the UK, where this study was conducted, or with the BALLETS study specifically.	This was sex as recorded on the original BALLETs data sheet so it would represent gender at the time of the study.<br>Whether we mention this is at editor discretion.
3b	Page 12, Table 1: Please clarify over what recall period is for the number of alcohol units consumed.	Likewise entered at the original data collection.

		We have added clarification to the table legend “consumed over post 6 months.”
4	Page 18, lines 8-16, “Results” section: “Having a BMI over 30 only slightly increased the probability of the liver outcome, although alcohol intake was more predictive.” I’m unclear what the authors mean by this. I would think that the authors are referring to Table 3 which shows hazard ratios, but a binary variable for BMI >30 is not used in this table. BMI >30 is referred to in Table 4 where the authors show posterior predicted probability of having a liver outcome at 5-, 10-, and 15-years post-baseline, but these results would not show comparative increase in predictability for alcohol intake. Further, Table 3 does not show that increased alcohol intake was more predictive than BMI, but shows that its 95% credible interval rules out a hazard=1 for having a liver outcome. At a minimum, these two statements should be separated and should reference their respective results tables.	Thank you. We have updated the text to better reflect the results, in particular highlighting that there is little evidence of a relationship between BMI and the liver outcome in either Table 3 or 4. We have also corrected the statements with regard to hazard ratios and separated statements, and referred to respective result Table.<br>“There was little evidence of a relationship between BMI and the probability of the liver outcome, which is also illustrated in Table 4. Alcohol intake was relatively more predictive of the liver outcome (HR 1.77; 1.12-2.67).”
5a	Page 20, Table 4: I believe this table has a typo, as you cannot calculate cumulative incidence for a “no primary outcome” group. I think the authors mean for these groups to be “no steatosis” vs “steatosis”.	This is correct – the column headings were mislabelled and have been amended.

5b	Page 20, Table 4: I'm unclear precisely what the authors mean by the last row of this table that reference BMI >30. Are they looking at posterior predictive probabilities for the subgroup of patients with BMI >30? Have they replaced the BMI and squared-BMI covariates with a binary indicator variable for BMI >30?	The fully adjustment model is estimated on the whole dataset, but we predict probabilities for the subgroup of patients with BMI>30. We have added a footnote to Table 4 and clarification in the methods section.<br>"* Posterior predictive probability among only those with a BMI>30 using the fully adjusted model estimated on the whole dataset."
**DISCUSSION**		
6a	"The probability of a liver end-point over ~15 years of follow-up is increased by about seven percentage points, adjusted for age and sex, decreasing to about six percentage points when also adjusted for obesity and alcohol intake. This is likely to reflect the effect of baseline obesity and alcohol on the explanatory variable (steatosis) and the subsequent effects of ongoing excess calorie and alcohol intake." It seems that the authors are implying that death due to increased BMI or alcohol intake act as competing risks for liver outcomes. However, on page 24 point 6 the authors explicitly state that they do not believe competing risks is an issue for this analysis. Please clarify both of these points.	The competing risks is an issue for Table 4 (cumulative incidence), but not Table 3 (hazard ratios). We thank the reviewer for this comment, which has prompted us to deal more comprehensively with the competing risks issue. We now add to limitations:<br>"Our hazard ratio estimand (Table 3) is a causal effect (the cause-specific HR is defined as the rate conditional on being alive and disease-free). The net risk (Table 4) gives a clinically

		useful measure of the rate of progression (admission or death for liver causes) but by estimating the net risk, meaning in the absence of competing risks, it overstates the absolute probability for people with substantial competing mortality, some of whom would die from a competing cause before a liver outcome occurs. In this cohort, non-liver deaths outnumbered liver outcomes by roughly four to one (381 vs 90-97) so the amount by which the probability is smaller is not trivial when taking competing risks into account. Published comparisons of a similar magnitude of competing events find over-estimates on the order of 15-20% (relative increase).(21) For younger and healthier cohorts with lower non-liver mortality, the difference from our net risk estimates would be smaller.”
6b	The discussion regarding what should be done when steatosis is found on a scan is somewhat complicated by the fact that the	We did acknowledge this issue under point 7 in what is now the strengths

	present analyses do not take into account what kinds of care patients received after steatosis was/was not found. It is possible that no preventative care was administered to those where steatosis was found, or that all patients with steatosis received as much intervening care as possible.	and limitations section. Here we note that probability of referral remains constant over time (Figure 4) and this is evidence against the idea that participants were referred when a positive scan was observed. One reason for selecting admissions as the primary outcome and attendances as secondary was to avoid the issue that the study observation of steatosis would prompt referral.
6c	Page 22, lines 33-41: The statement that the findings of this manuscript are consistent with recent Lancet consensus guidelines conflates the question being discussed up until this point (“what should we do when we find steatosis?”) with their second proposed question (“should we actively look for [steatosis]?”). This statement should be moved to the latter part of the section where they discuss the appropriate question it references.	While we do not think our findings are of no relevance to the active looking issue we have now removed this section in light of comments from Reviewer 1.
6d	To this point, it seems that the question “should we actively look for [steatosis]?” has already been answered by consensus guidelines and thus discussion on this point is moot.	Now covered by previous point

6e	Pages 23-25: I am unclear what purpose the numbered points attempt to serve, as they appear to be a random collection of leftover thoughts. Without contextualization, I take issue with several of them, particularly point 1 (justification and context needed for inclusion of this new outcome, particularly if to be included in the primary composite), point 2 (unclear if risk of steatosis also varies based on different global populations), point 5 (unclear the point the authors are attempting to make), and point 6 (discussed in my comment 6a) . If the authors wish to integrate these into the discussion they need to be appropriately synthesized and contextualized.	Now rearranged and re-ordered as per recommendations from Reviewer 1.

VERSION 2 - REVIEW

**Reviewer**	**1**
**Name**	**Jarvis, Helen**
**Affiliation**	**Newcastle University, Institute of Health & Society**
**Date**	**14-Sep-2026**
**COI**	

points from my initial review have been adequately addressed.

Thanks

Very minor suggestion - would suggest changing 'family doctor' to 'primary care practitioner' at the top of page 26 in m

**Reviewer**	**2**
**Name**	**Ryan Baumann, Mary**

Affiliation **University of Wisconsin-Madison School of Medicine and Public Health, Population Health Sciences; Biostatistics and Medical Informatics**

Date **01-Oct-2026**

COI

I thank the authors for addressing many of my comments from the first review. However, there are still a few remaining point that I believe should be addressed. I've listed them below in order of where they appear in the manuscript.

1. Page 11, lines 13-18: In your section regarding the secondary analysis using an alternatively-defined outcome, I would clarify that hypothesis tests were not conducted on these alternatively-defined groups to avoid p-hacking but that statistical analyses were in fact done to estimate effect sizes and compare on magnitude to the primary analysis.
    a. As an aside, I don't believe what the authors are describing, even if they did perform hypothesis tests, would constitute p-hacking. I believe p-hacking would happen if you were to perform these analyses and then selectively report results based on p-values. If you state your reason for the purpose of the analyses, perform the analyses, report the results regardless of p-value, and state (in)consistency with the primary analysis, I believe that would that just be a sensitivity analysis. However, this is merely an observation on my part, and the authors do not need to address this part of the comment.
2. Page 12, lines 18-21: I would be explicit about which variables serve as confounders and which serve as variables of clinical interest; since I could convince myself that all 4 variables could be considered confounders, I'd appreciate the clarification.
3. Pages 12-13: When describing the baseline characteristics, could you include your cutoff definitions of overweight/obese for BMI and recommended alcohol units per week since they don't map exactly to a single category displayed in the table? I can back-calculate for BMI fairly easily, but it's a bit more labor-intensive with the alcohol units.
4. Page 18 line 18, and Supplementary Material 10: You use the terms "fatty" and "not fatty" groups here. I would align the terminology here with what you use in the rest of the main text.
5. Table 3: I believe the label of "primary outcome" is meant to represent Steatosis group – perhaps the label should be changed to "exposure"?
6. Page 26, point 9: I don't think the results you've reported constitute causal effects since you've not provided a directed acyclic graph (DAG; or equivalent) showing that you've closed all the backdoor pathways, nor have you discussed satisfaction of the usual causal assumptions. Table 3 provides associative relative effects, while I agree with the assessment of Table 4 as being useful to evaluate absolute
differences. I don't want this comment to be construed as arguing that the estimates the authors report aren't informative – I believe they absolutely are –

just that I believe further causal modeling strategies would need to be employed to produce true causal effects.

7. Page 27, lines 24-27: I wouldn't say that steatosis doubles the risk of liver outcomes, but that it's associated with a doubling in the risk. I don't think you've established whether steatosis is the mechanism or a symptom of the mechanism; however, I am not a subject expert and defer to the editor and other reviewer on this matter.

VERSION 2 - AUTHOR RESPONSE

Reviewer 1

#	Reviewer Comment	Authors' Response
1	points from my initial review have been adequately addressed. Thanks. Very minor suggestion - would suggest changing 'family doctor' to 'primary care practitioner' at the top of page 26 in m	Thank you, amendment made.

Reviewer 2

#	Reviewer Comment	Authors' Response
1	Page 11, lines 13-18: In your section regarding the secondary analysis using an alternatively-defined outcome, I would clarify that hypothesis tests were not conducted on these alternatively-defined groups to avoid p-hacking but that statistical analyses were in fact done to estimate effect sizes and compare on magnitude to the primary analysis.	We have made this adjustment.
1a	As an aside, I don't believe what the authors are describing, even if they did perform hypothesis tests, would constitute p-hacking. I believe p-hacking would happen if you were to perform these analyses and then	I have looked it up – it is "trying different ways of analysis until you get a significant result and then repeating it as though the analysis had been planned in advance."

	selectively report results based on p-values. If you state your reason for the purpose of the analyses, perform the analyses, report the results regardless of p-value, and state (in)consistency with the primary analysis, I believe that would that just be a sensitivity analysis. However, this is merely an observation on my part, and the authors do not need to address this part of the comment.	
2	Page 12, lines 18-21: I would be explicit about which variables serve as confounders and which serve as variables of clinical interest; since I could convince myself that all 4 variables could be considered confounders, I'd appreciate the clarification.	We have clarified the point: "…and then including BMI and alcohol (to examine the association between steatosis and alcohol, net of these factors)."
3	Pages 12-13: When describing the baseline characteristics, could you include your cutoff definitions of overweight/obese for BMI and recommended alcohol units per week since they don't map exactly to a single category displayed in the table? I can back-calculate for BMI fairly easily, but it's a bit more labor-intensive with the alcohol units.	Clarification added "…overweight (25-29.99kg/m^2) or obese (>30kg/m^2)…" "…recommended alcohol units per week (14 units/week)…"
4	Page 18 line 18, and Supplementary Material 10: You use the terms "fatty" and "not fatty" groups here. I would align the terminology here with what you use in the rest of the main text.	Terminology changed in text and in Supplementary Materials, thank you.

5	Table 3: I believe the label of "primary outcome" is meant to represent Steatosis group – perhaps the label should be changed to "exposure"?	No, the hazard is that of having the outcome as defined.
6	Page 26, point 9: I don't think the results you've reported constitute causal effects since you've not provided a directed acyclic graph (DAG; or equivalent) showing that you've closed all the backdoor pathways, nor have you discussed satisfaction of the usual causal assumptions. Table 3 provides associative relative effects, while I agree with the assessment of Table 4 as being useful to evaluate absolute differences. I don't want this comment to be construed as arguing that the estimates the authors report aren't informative – I believe they absolutely are – just that I believe further causal modeling strategies would need to be employed to produce true causal effects.	We have changed "causal" to "associative relative" effect.
7	Page 27, lines 24-27: I wouldn't say that steatosis doubles the risk of liver outcomes, but that it's associated with a doubling in the risk. I don't think you've established whether steatosis is the mechanism or a symptom of the mechanism; however, I am not a subject expert and defer to the editor and other reviewer on this matter.	Change made. Many thanks for careful reading of our paper.
